# Characterization of Atrial Propagation Patterns and Fibrotic Substrate With a Modified Omnipolar Electrogram Strategy in Multi-Electrode Arrays

**DOI:** 10.3389/fphys.2021.674223

**Published:** 2021-09-03

**Authors:** Jennifer Riccio, Alejandro Alcaine, Sara Rocher, Laura Martinez-Mateu, Sergio Laranjo, Javier Saiz, Pablo Laguna, Juan Pablo Martínez

**Affiliations:** ^1^Biomedical Signal Interpretation and Computational Simulation Group, Aragón Institute of Engineering Research, IIS Aragón, Universidad de Zaragoza, Zaragoza, Spain; ^2^Facultad de Ciencias de la Salud, Universidad San Jorge, Zaragoza, Spain; ^3^Centro de Investigación Biomédica en Red en Bioingeniería, Biomateriales y Nanomedicina, Zaragoza, Spain; ^4^Centro de Investigación e Innovación en Ingeniería, Universitat Politècnica de València, Valencia, Spain; ^5^Departamento de Teoría de la Señal y Comunicaciones y Sistemas Telemáticos y Computación, Universidad Rey Juan Carlos, Madrid, Spain; ^6^Department of Pediatric Cardiology, Hospital Santa Marta, Centro Hospitalar de Lisboa Central, Lisbon, Portugal

**Keywords:** atrial fibrosis, atrial fibrallation, multi-electrode array, unipolar electrograms, bipolar electrograms, omnipolar electrogram, modified omnipolar electrogram, conduction velocity

## Abstract

**Introduction:** The omnipolar electrogram method was recently proposed to try to generate orientation-independent electrograms. It estimates the electric field from the bipolar electrograms of a clique, under the assumption of locally plane and homogeneous propagation. The local electric field evolution over time describes a loop trajectory from which omnipolar signals in the propagation direction, substrate and propagation features, are derived. In this work, we propose substrate and conduction velocity mapping modalities based on a modified version of the omnipolar electrogram method, which aims to reduce orientation-dependent residual components in the standard approach.

**Methods:** A simulated electrical propagation in 2D, with a tissue including a circular patch of diffuse fibrosis, was used for validation. Unipolar electrograms were calculated in a multi-electrode array, also deriving bipolar electrograms along the two main directions of the grid. Simulated bipolar electrograms were also contaminated with real noise, to assess the robustness of the mapping strategies against noise. The performance of the maps in identifying fibrosis and in reproducing unipolar reference voltage maps was evaluated. Bipolar voltage maps were also considered for performance comparison.

**Results:** Results show that the modified omnipolar mapping strategies are more accurate and robust against noise than bipolar and standard omnipolar maps in fibrosis detection (accuracies higher than 85 vs. 80% and 70%, respectively). They present better correlation with unipolar reference voltage maps than bipolar and original omnipolar maps (Pearson's correlations higher than 0.75 vs. 0.60 and 0.70, respectively).

**Conclusion:** The modified omnipolar method improves fibrosis detection, characterization of substrate and propagation, also reducing the residual sensitivity to directionality over the standard approach and improving robustness against noise. Nevertheless, studies with real electrograms will elucidate its impact in catheter ablation interventions.

## 1. Introduction

Diagnosis and treatment of a wide range of atrial arrhythmias, including atrial fibrillation, require extraction of substrate and propagation features from intracardiac electrograms (EGMs) (Houben and Alessie, 2006). Mechanisms underlying atrial fibrillation are not yet completely clear (Calkins et al., 2017) and no single-one can comprehensively explain the arrhythmia. The electrical isolation of focal trigger sites in the pulmonary veins (Keane and Ruskin, 2002) may abrogate early stage atrial fibrillation when anti-arrhythmic drug therapy becomes ineffective (Wazni et al., 2005). However, in more advanced stages of persistent atrial fibrillation, additional arrythmogenic substrate and mechanisms such as fibrosis are likely present (Lau et al., 2017), ablation of which was shown to improve success rates in patients (Jadidi et al., 2016).

Due to its unstable nature from beat to beat, atrial fibrillation is better investigated through simultaneous EGMs, recorded by high-density multi-electrode catheters from multiple sites at the same time, rather than by analyzing sequential EGMs, each recorded at a different time within the mapping procedure. From such signals, meaningful features for characterizing atrial substrate and propagation pattern are extracted and mapped through 3D electroanatomic mapping systems, thus helping physicians to visualize the locations that are generating the erratic propagation.

Characterization of atrial substrate is typically performed by mapping the peak-to-peak amplitude of bipolar EGMs (b-EGMs). This strategy identifies as fibrosis (Magtibay et al., 2019) those areas having voltage lower than 0.5 mV (Sim et al., 2019). The fibrotic areas determined in this way constitute a potential substrate for atrial fibrillation maintenance (Burstein and Nattel, 2008) and, consequently, a target for ablation, in combination with pulmonary veins isolation (Haldar et al., 2017). However, the dependence of b-EGMs on multiple factors, including catheter orientation with respect to wavefront direction, electrode size, inter-electrode spacing and contact with the tissue, affect bipolar amplitude values (Magtibay et al., 2019). Moreover, the proposed threshold to define low-voltage areas has not received histological or electrophysiological evidence (Yamaguchi et al., 2019).

In current practice, characterization of atrial propagation patterns is performed by assessing local activation times (Magtibay et al., 2019), using unipolar EGMs (u-EGMs) or b-EGMs. However, both u-EGMs and b-EGMs present limitations because: (1) u-EGMs are sensitive to electric far-field disturbances due to other large cardiac structures such as the ventricles; (2) b-EGMs depend on the angle between the catheter and wavefront propagation direction; (3) both are sensitive to local recording noise.

The recently proposed *Omnipolar EGM* (OP-EGM) method tries to cope with the problems affecting unipolar and bipolar measurements (Deno et al., 2017). It is based on the estimation of the electric field from all the b-EGMs locally recorded at each group of nearby electrodes, referred as *clique* by Deno et al. (2017), under the assumption of locally plane and homogeneous propagation within the clique. Two types of cliques are used in this work: (a) square cliques, which consist of four-electrode sets forming a square, and (b) triangular cliques, which consist of three-electrode sets formed by an electrode and two adjacent ones, one in each direction of the 2D plane. From these electrode sets, omnipolar signals and parameters are derived and associated to a single point virtually located at the center of the clique. The electric field evolution over time within each clique describes a loop which depends on the propagation direction. OP-EGM signals are obtained as projections of this loop onto the propagation direction, as if a virtual bipole in that direction had been used. In this way, the dependence on the catheter orientation is theoretically reduced. Substrate and propagation parameters such as peak-to-peak amplitude of OP-EGM signals and conduction velocity (CV) are also estimated at the clique level without requiring previous estimation of local activation times.

In this work, we proposed voltage and CV mapping strategies based on modifications of the OP-EGM method (MOP-EGM) by using two clique configurations: square and triangular cliques. These modifications are hypothesized to improve the accuracy and robustness of the voltage and CV estimates and to reduce the error induced by the b-EGMs dependence on catheter orientation, which is not fully compensated by the OP-EGM approach. Specifically, the aims of this study are: (1) to assess the estimates of CV and propagation direction within each clique with the proposed MOP-EGM method as compare to the ones obtained by the original OP-EGM; (2) to propose mapping strategies based on omnipolar EGMs estimations to characterize the atrial substrate; and (3) to assess the ability of the proposed maps in detecting a fibrotic patch and in reproducing unipolar voltage maps.

## 2. Methods

### 2.1. 2D Atrial Sheet Model

In order to assess the proposed mapping strategies, a 2D atrial tissue of 4 × 4 cm under chronic atrial fibrillation (cAF) conditions was simulated by dividing it into adjacent square elements whose centers were separated 0.1 mm. Within the tissue slice, a circular patch with a diameter of 2 cm was defined, whose center was at the center of the 2D tissue. Inside this circular patch, a diffuse fibrosis pattern was randomly defined following a uniform distribution. All the non-fibrotic nodes were assigned the Courtemanche cellular model stabilized during 1 min at basic cycle length (BCL) 500 ms (Courtemanche et al., 1998). To reproduce left atrial action potential (AP) morphology, the maximum ionic conductance of the rapid delayed rectifier potassium current (*I*_*Kr*_) was selected 1.6-fold greater than the original right atrial model (Li et al., 2001; Martinez-Mateu et al., 2018). Additionally, electrical remodeling induced by cAF was introduced through the variation of the maximum conductances of the transient outward potassium current (*I*_*to*_), the L-type calcium current (*I*_*CaL*_), the inward rectifier potassium current (*I*_*K*1_), the ultrarapid outward potassium current (*I*_*Kur*_) and the slow delayed rectifier potassium current (*I*_*Ks*_) (see Supplementary Table 1), as in previous computational studies (Tobón et al., 2013; Martinez-Mateu et al., 2018). Twenty percent of the nodes within the fibrotic patch were assigned the Maleckar fibroblast model (Maleckar et al., 2009). The intercellular conduction velocity was decreased to 30% in all elements with at least one fibroblast node (Sánchez et al., 2019). Although the percentage of atrial fibrosis is very patient-dependent, the 20% represents the threshold value between stage II and stage III according to the Utah classification (Chelu et al., 2018), therefore being a realistic percentage for this study. Supplementary Figure 1 shows the effect of adding the cAF electrical remodeling and the fibroblast coupling in the Courtemanche model. The APs were registered in two different myocytes from the mesh, one outside the fibrotic patch and the other inside the fibrotic patch coupled with two fibroblasts. Electrical remodeling produces a 55% reduction in duration measured at 90% repolarization (*APD*_90_) (248 vs. 111 ms), in concordance with experimental data (Bosch et al., 1999). Fibroblast coupling with cAF myocytes makes resting potential less negative (83 vs. 78 mV) and elongates the *APD*_90_ (111 vs. 120 ms). Simulations were run in ELVIRA software (Heidenreich et al., 2010). The monodomain formulation was solved using the operator splitting numerical scheme with a constant time step *dt* = 0.01 ms and a spatial resolution *dx* = 0.1 mm.

**Figure 1:**
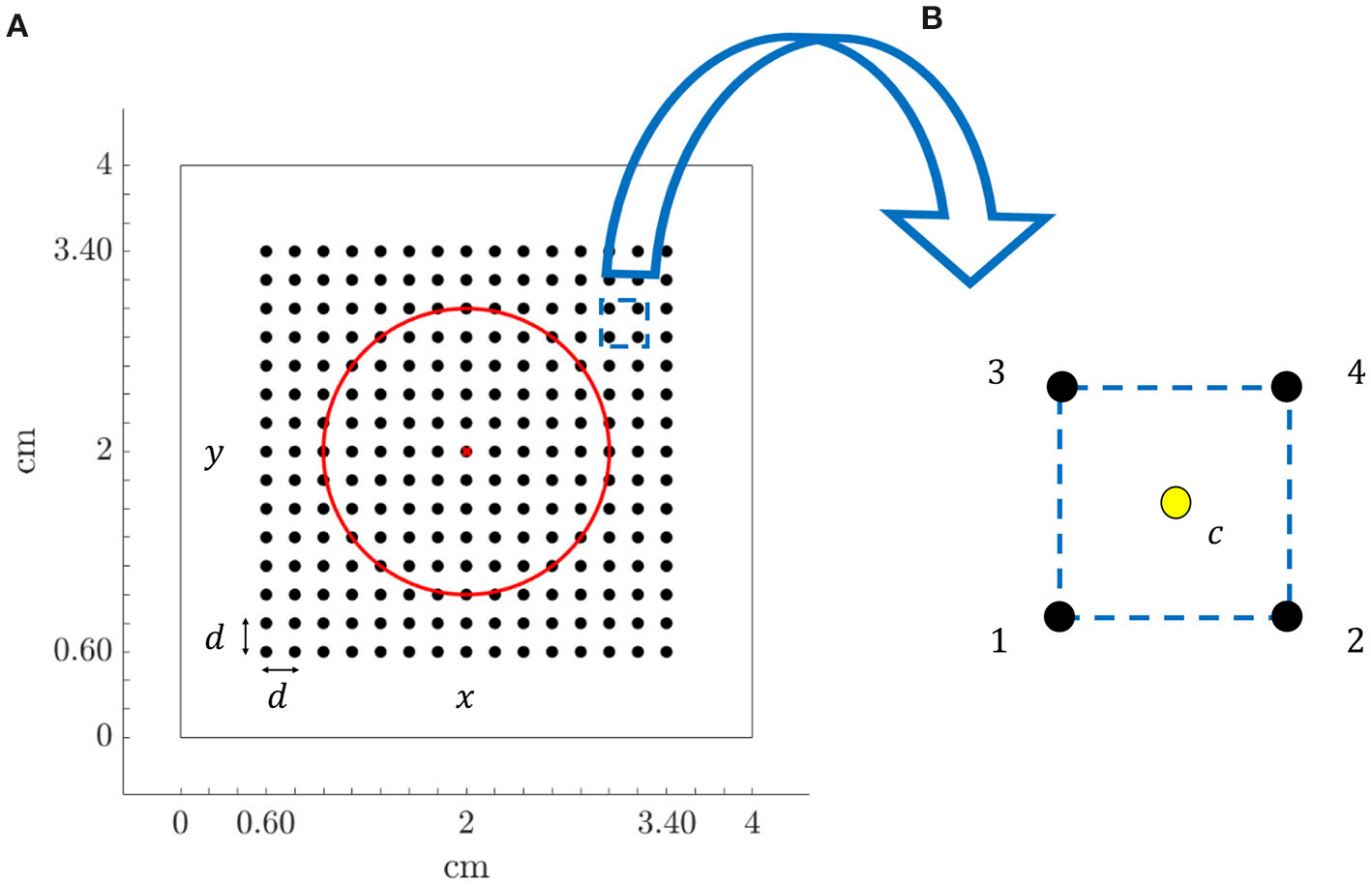
**(A)** Tissue geometry (4 × 4 cm) with overprinted the *N* × *N* (*N* = 15) MEA electrodes (black dots) and inter-electrode spacing *d* = 2 mm, in the 2D Cartesian coordinate system defined by *x* and *y*. Red circle encompasses fibrotic tissue area and red cross represents the electrode located at the center of the tissue. **(B)** Arrangement of four electrodes (clique) from the MEA, internally denoted with 1, 2, 3, and 4, where location at the centre of the square (yellow dot) is indicated with letter *c*.

Recording electrodes were distributed over the simulated tissue, mimicking a *N* × *N* high-density multi-electrode array (MEA) (*N* = 15), where the inter-electrode distance was *d* = 2 mm. The simulated tissue geometry, including the MEA location and the fibrotic patch, is shown in Figure 1A. Figure 1B depicts a generic square clique. To keep the same simplified notation in each position within the MEA, its lower left electrode, corresponding to location (*i, j*) within the MEA, is referred to as electrode 1. The rest electrodes of the clique are numbered from left to right and bottom to top. Therefore, electrodes 2, 3, and 4 correspond to locations (*i* + 1, *j*), (*i, j* + 1), and (*i* + 1, *j* + 1), respectively.

**Figure 2:**
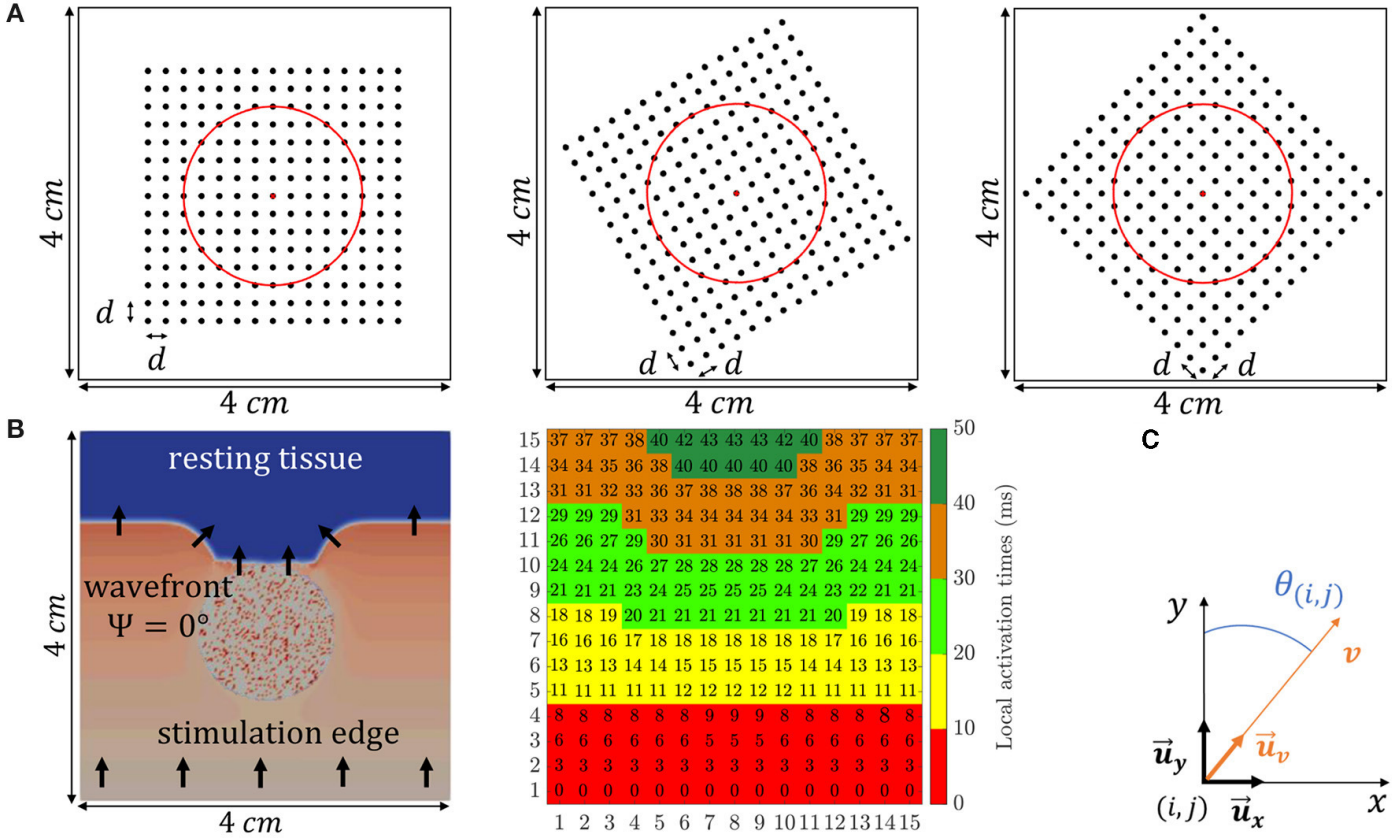
**(A)** The three different MEA orientations with respect to the tissue considered in this study: Ψ = 0° (leftmost), Ψ = 30° (middle) and Ψ = 45° (rightmost). (**B**, leftmost) Activation distribution at a particular time instant, where black arrows indicate propagating wave direction. (**B**, rightmost) Local activation times map representing the propagation pattern. **(C)** Local 2D Cartesian coordinate system defined by unit vectors ${\vec{\text{u}}}_{x}$ and ${\vec{\text{u}}}_{y}$.

The simulated electrode grid is located so that its central electrode corresponds to the center of both the tissue slice and the fibrotic patch, and the MEA is rotated by an angle Ψ with respect to the tissue fiber orientation. Three different MEA orientations with respect to the tissue preferential direction were considered: Ψ = 0°, Ψ = 30°, and Ψ = 45°, which are illustrated in Figure 2A. For the case when Ψ = 0°, the propagating wavefront direction was depicted in Figure 2B, leftmost, where the fibrotic patch at the center of the tissue is also marked with a diffuse colored area. Moreover, for the same case, the propagation pattern was shown in Figure 2B, rightmost, by means of the map of local activation times, estimated as the maximal negative slope of the available synthetic u-EGMs (Paul et al., 1990). A video content showing propagating wavefront can also be found in the Supplementary Material.

The MEA grid was placed at *z* = 1 mm distance from the tissue surface. This represents a plausible compromise between recording realistic amplitude EGMs and the real clinical setting situation where there is no guarantee of maintaining a perfect electrode-tissue contact during the mapping.

### 2.2. Synthetic Signals

Unipolar EGMs *u*_*i, j*_(*t*) were simulated as generated by the propagation wave when passing by electrodes located at sites (*i, j*), (*i* ∈ {1, …, *N*}, *j* ∈ {1, …, *N*}) of the grid. They were computed in a volumetric tissue-blood model with a temporal resolution of 1 ms, as in Martinez-Mateu et al. (2019), by using an approximation of the bidomain formulation in two steps (Keller et al., 2010; Martinez-Mateu et al., 2018). By assuming equal anisotropy ratios for the intracellular and extracellular conductance tensors, bidomain equations can be decoupled accounting one of them for changes in the transmembrane potential and the other one for changes in the extracellular potential. Therefore, in a first step, transmembrane potential was solved by the monodomain approach. Then, in a second step, the already calculated transmembrane potential was used to obtain the extracellular potential, considering the tissue immersed in a non-conductive bath.

Simulated signals were obtained with a sampling frequency of 1 kHz, and had a duration of 0.5 s, containing one single activation (depolarization plus repolarization). Bipolar EGMs $b_{i,j}^{x}\left(t\right)$ and $b_{i,j}^{y}\left(t\right)$ were derived along the two MEA directions as follows:

(1) $$\begin{array}{l} b_{i,j}^{x}\left(t\right)=u_{i+1,j}\left(t\right)-u_{i,j}\left(t\right);i\in \left\{1,...,N-1\right\},\;j\in \left\{1,...,N\right\}\\ b_{i,j}^{y}\left(t\right)=u_{i,j+1}\left(t\right)-u_{i,j}\left(t\right);i\in \left\{1,...,N\right\},j\in \left\{1,...,N-1\right\}. \end{array}$$

Bipolar signals, as defined in Equation (1), are used to compute bipolar voltage maps for performance comparison, as described in subsections 2.12, 2.13.

The omnipolar methodological developments assume a locally plane wave propagation within a clique, with a velocity vector $\text{v}=v{\vec{\text{u}}}_{v}$, where ${\vec{\text{u}}}_{v}$ denotes a unit direction vector whose spatial dependence has been omitted for notation simplicity. In the 2D Cartesian coordinate system defined by the unit vectors in the main directions of the MEA (${\vec{\text{u}}}_{x}$ and ${\vec{\text{u}}}_{y}$), the local plane wave propagation direction within the clique having electrode (*i, j*) in its lower left corner, ${\vec{\text{u}}}_{v}$, forms an angle θ_(*i, j*)_ with the direction of ${\vec{\text{u}}}_{y}$, as illustrated in Figure 2C. It must be noted that the angle Ψ is unique for each 2D catheter configuration and should not be mistaken for θ_(*i, j*)_, which depends on local propagation at (*i, j*) electrode position, see black arrows direction variations in Figure 2B, leftmost.

Examples of unipolar and bipolar EGMs used in this study, corresponding to the case when the global propagation direction of the wave and MEA direction form an angle Ψ = 0°, are shown in Supplementary Figure 2. In that case, b-EGMs are reported with added noise as well (see also subsection 2.11).

### 2.3. Activation Times and Relation Between Electrograms Under Plane Wave Assumption

Let us consider any four-electrode clique within the MEA, like the one in Figure 1B. Let *t*_*c*_ be the reference time at which the center *c* of the selected square is activated. Assuming constant velocity within the arrangement, the activation times at the four electrodes are:

(2) $$\begin{array}{l} t_{1}=t_{c}-\frac{d}{2v}\sin\theta -\frac{d}{2v}\cos\theta ;\;t_{2}=t_{c}+\frac{d}{2v}\sin\theta -\frac{d}{2v}\cos\theta ;\\ t_{3}=t_{c}-\frac{d}{2v}\sin\theta +\frac{d}{2v}\cos\theta ;\;t_{4}=t_{c}+\frac{d}{2v}\sin\theta +\frac{d}{2v}\cos\theta . \end{array}$$

Let ϕ(*t*) be the unipolar voltage waveform generated by a plane wave when passing by a given position at time *t* = 0. When one or more electrodes within the square of Figure 1B are activated by ϕ(*t*) passage, the following u-EGMs and b-EGMs are modeled as:

(3) $$\begin{array}{l} u_{m}\left(t\right)=\phi \left(t-t_{m}\right),m\in \left\{1,2,3,4\right\} \end{array}$$

(4) $$\begin{array}{l} b_{mn}\left(t\right)=u_{n}\left(t\right)-u_{m}\left(t\right)=\phi \left(t-t_{n}\right)-\phi \left(t-t_{m}\right),\\ \;m,n\neq m\in \left\{1,2,3,4\right\}. \end{array}$$

It should be noted that bipolar signals in Equation (4) are derived within each clique along *x* direction (*b*_12_(*t*) and *b*_34_(*t*)), *y* direction (*b*_13_(*t*) and *b*_24_(*t*)), as well as along the directions of its diagonals (*b*_14_(*t*) and *b*_23_(*t*)). Depending on the waveform arrival time to each electrode *t*_*m*_, the activation time of each b-EGM *b*_*mn*_(*t*) corresponds to the passing of the wave by the middle point between electrodes *m* and *n*. Therefore, relative delays (misalignment) occur between the different *b*_*mn*_(*t*).

By using the Taylor's series expansion of *u*_*m*_(*t*) = ϕ(*t* − *t*_*m*_) around *t*_*m*_ = *t*_*c*_, we can approximate:

(5) $$\begin{array}{ll} u_{m}\left(t\right) & ≅\phi \left(t-t_{c}\right)-\Delta t_{m}\phi^{\prime }\left(t-t_{c}\right)+\frac{1}{2}{\left(\Delta t_{m}\right)}^{2}\phi^{\prime \prime }\left(t-t_{c}\right) \end{array}$$

(6) $$\begin{array}{l} b_{mn}\left(t\right)≅-\Delta t_{mn}\phi^{\prime }\left(t-t_{c}\right)\\ \;+\frac{1}{2}\left({\left(\Delta t_{n}\right)}^{2}-{\left(\Delta t_{m}\right)}^{2}\right)\phi^{\prime \prime }\left(t-t_{c}\right), \end{array}$$

where Δ*t*_*mn*_ = *t*_*n*_−*t*_*m*_ are the differences between activation times at electrodes *m* and *n*, *m, n* ∈ {1, 2, 3, 4}, and Δ*t*_*m*_ = *t*_*m*_−*t*_*c*_ are the differences between activation time at electrode *m* and the reference activation time of the center of the square. By replacing Equation (2) in Equation (6) and operating, the following b-EGMs expressions are derived:

(7) $$\begin{array}{l} b_{12}\left(t\right)≅-\frac{d}{v}\sin\theta \phi^{\prime }\left(t-t_{c}\right)-\frac{d^{2}}{4v^{2}}\sin\left(2\theta \right)\phi^{\prime \prime }\left(t-t_{c}\right)\\ \;≅\;-\frac{d}{v}\sin\theta \phi^{\prime }\left(t-t_{c}+\frac{d}{2v}\cos\left(\theta \right)\right) \end{array}$$

(8) $$\begin{array}{l} b_{34}\left(t\right)≅-\frac{d}{v}\sin\theta \phi^{\prime }\left(t-t_{c}\right)+\frac{d^{2}}{4v^{2}}\sin\left(2\theta \right)\phi^{\prime \prime }\left(t-t_{c}\right)\\ \;≅\;-\frac{d}{v}\sin\theta \phi^{\prime }\left(t-t_{c}-\frac{d}{2v}\cos\left(\theta \right)\right) \end{array}$$

(9) $$\begin{array}{l} b_{13}\left(t\right)≅-\frac{d}{v}\cos\theta \phi^{\prime }\left(t-t_{c}\right)-\frac{d^{2}}{4v^{2}}\sin\left(2\theta \right)\phi^{\prime \prime }\left(t-t_{c}\right)\\ \;≅\;-\frac{d}{v}\cos\theta \phi^{\prime }\left(t-t_{c}+\frac{d}{2v}\sin\left(\theta \right)\right) \end{array}$$

(10) $$\begin{array}{l} b_{24}\left(t\right)≅-\frac{d}{v}\cos\theta \phi^{\prime }\left(t-t_{c}\right)+\frac{d^{2}}{4v^{2}}\sin\left(2\theta \right)\phi^{\prime \prime }\left(t-t_{c}\right)\\ \;≅\;-\frac{d}{v}\cos\theta \phi^{\prime }\left(t-t_{c}-\frac{d}{2v}\sin\left(\theta \right)\right) \end{array}$$

(11) $$\begin{array}{l} b_{14}\left(t\right)≅\;-\left(\frac{d}{v}\sin\theta +\frac{d}{v}\cos\theta \right)\phi^{\prime }\left(t-t_{c}\right)\\ \;=-\frac{\sqrt{2}d}{v}\cos\left(\theta -45^{{}^{\circ}}\right)\phi^{\prime }\left(t-t_{c}\right) \end{array}$$

(12) $$\begin{array}{l} b_{23}\left(t\right)≅\;+\left(\frac{d}{v}\sin\theta -\frac{d}{v}\cos\theta \right)\phi^{\prime }\left(t-t_{c}\right)\\ \;=+\frac{\sqrt{2}d}{v}\sin\left(\theta -45^{{}^{\circ}}\right)\phi^{\prime }\left(t-t_{c}\right). \end{array}$$

From these expressions we can relate the second order approximations of the different pairs of b-EGMs *b*_*mn*_(*t*), showing the previously mentioned delay between the different bipolar signals. If we relate *b*_12_(*t*) and *b*_13_(*t*), measured in different *x* and *y* directions, we obtain:

(13) $$\begin{array}{l} b_{13}\left(t\right)≅-\frac{d}{v}\cos\theta \phi^{\prime }\left(t-t_{c}\right)-\frac{d^{2}}{4v^{2}}\sin\left(2\theta \right)\phi^{\prime \prime }\left(t-t_{c}\right)\\ \;≅\frac{\cos\theta }{\sin\theta }\;b_{12}\left(t-\frac{d}{v}\frac{\sin\left(45^{{}^{\circ}}-\theta \right)}{\sqrt{2}}\right). \end{array}$$

Equation (13) shows that the b-EGM along *y*-direction (1–3) is a scaled version of the b-EGM along *x*-direction (1–2) by a factor $\left(\frac{\cos\theta }{\sin\theta }\right)$ and delayed by a time $\tau =\frac{d}{v}\frac{\sin\left(45^{{}^{\circ}}-\theta \right)}{\sqrt{2}}$. This corresponds to the delay between the pass of the wave by the middle points of each electrode pair, separated $d/\sqrt{2}$. As expected, for θ = 90° (propagation in *x*-direction), *b*_13_(*t*) = 0, while for θ = 45°, the delay is τ = 0, meaning that both bipolar EGMs in the *x*- and *y*- directions are activated at the same time. Similarly, if we relate *b*_12_(*t*) with *b*_34_(*t*), both along the *x*-direction, we obtain:

(14) $$\begin{array}{l} b_{34}\left(t\right)≅-\frac{d}{v}\sin\theta \phi^{\prime }\left(t-t_{c}\right)+\frac{d^{2}}{4v^{2}}\sin\left(2\theta \right)\phi^{\prime \prime }\left(t-t_{c}\right)\\ \;≅b_{12}\left(t-\frac{d}{v}\cos\theta \right). \end{array}$$

Equation (14) shows that b-EGMs along parallel directions have the same amplitudes (as expected), and the delay $\tau =\frac{d}{v}\cos\theta$ ranges from 0, when the propagation is in the same direction than the electrode pairs (θ = 90°), which are therefore activated at the same time, to a maximum of *d*/*v*, when the propagation is orthogonal to the direction of the electrode pairs (θ = 0°).

### 2.4. OP-EGM Framework

The purpose of the OP-EGM method is to obtain EGM amplitude and propagation features which are invariant to the relative angle between the propagation direction and the catheter (Deno et al., 2017). This method is based on the relationship between the spatial gradient of voltage ϕ(*t*), and the electric field (E-field) at the extracellular-myocardial interface. Let us consider a locally plane myocardial surface, defined by its normal unit vector ${\vec{\text{u}}}_{n}$. At each point of the surface, the 3D electric field is described in the right-handed coordinate system defined by the normal unit vector ${\vec{\text{u}}}_{n}$, the unit vector in the propagation direction ${\vec{\text{u}}}_{p}$ and in the direction orthogonal to ${\vec{\text{u}}}_{p}$ within the 2D plane ${\vec{\text{u}}}_{⊥}$, see Deno et al. (2017).

**Figure 3:**
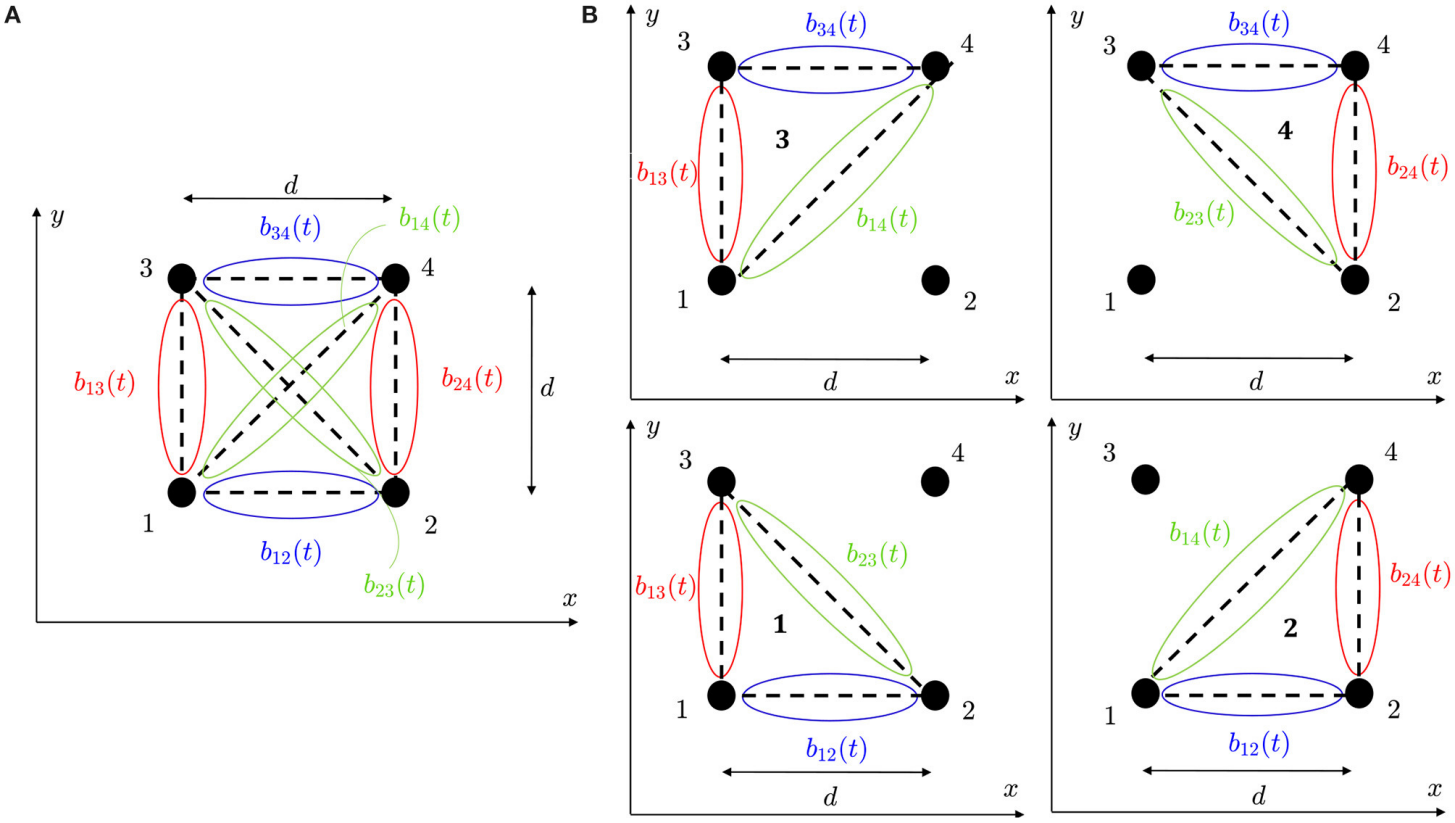
**(A)** Four-electrode clique. **(B)** Three-electrode cliques.

Since a 2D MEA was used in this study, only the electric field components in the plane defined by ${\vec{\text{u}}}_{p}$ and ${\vec{\text{u}}}_{⊥}$ were estimated: $\text{E}\left(t\right)={\text{E}}_{p}\left(t\right){\vec{\text{u}}}_{p}+{\text{E}}_{⊥}\left(t\right){\vec{\text{u}}}_{⊥}$, with $\text{E}\left(t\right)={\left[\begin{array}{cc} E_{x}\left(t\right) & E_{y}\left(t\right) \end{array}\right]}^{T}$. The traveling wave is assumed to be locally plane and homogeneous within each clique of the MEA. Under that assumption, the relation between the local electric field and the measured b-EGMs is given by the following linear system:

(15) $$\begin{array}{l} b_{\varrho }\left(t\right)=-{\text{D}}_{\varrho }^{T}E\left(t\right), \end{array}$$

where ***b***(*t*) is a vector containing the available b-EGMs, **D** is a matrix of interelectrode distances, and ϱ ∈ {_□_,_△_} denotes the clique type, square (_□_) or triangular (_△_), respectively. In case of square clique, as the one illustrated in Figure 3A, ***b***_ϱ_(*t*) ≡ ***b***__□__(*t*) contains six b-EGMs, ${\text{b}}_{□}\left(t\right)={\left[\begin{array}{cccccc} b_{12}\left(t\right) & b_{13}\left(t\right) & b_{14}\left(t\right) & b_{34}\left(t\right) & b_{24}\left(t\right) & b_{23}\left(t\right) \end{array}\right]}^{T}$ and **D**_ϱ_ ≡ **D**__□__ is a 2 × 6 matrix, reported below. Similarly, in case of triangular configurations, as the clique 1 in Figure 3B, ***b***_ϱ_(*t*) ≡ ***b***__△, 1__(*t*) contains three b-EGMs, ${\text{b}}_{△,1}\left(t\right)={\left[\begin{array}{ccc} b_{12}\left(t\right) & b_{13}\left(t\right) & b_{23}\left(t\right) \end{array}\right]}^{T}$ and **D**_ϱ_ ≡ **D**__△, 1__ is a 2 × 3 matrix, also shown below.

(16) $$\begin{array}{l} {\text{D}}_{□}=\left[\begin{array}{cccccc} -d & 0 & -d & -d & 0 & +d\\ 0 & -d & -d & 0 & -d & -d \end{array}\right],\;{\text{D}}_{△,1}=\left[\begin{array}{ccc} -d & 0 & +d\\ 0 & -d & -d \end{array}\right]. \end{array}$$

Expressions similar to **D**__△, 1__ were obtained for the other three-electrode configurations of the clique shown in Figure 3B. Note that with this formulation, the electric field is taken at the clique center, assuming that there are no delays between b-EGM components.

From Equation (15) we see that the projection of the E-field along the inter-electrode distance results in the corresponding b-EGM. Since measured b-EGMs will be affected by noise and model mismatch errors, we can use the least-squares criterion to estimate the electric field ${\hat{\text{E}}}_{\varrho }\left(t\right)$ at the center of each clique:

(17) $$\begin{array}{l} {\hat{E}}_{\varrho }\left(t\right)=-{\left({\text{D}}_{\varrho }{\text{D}}_{\varrho }^{T}\right)}^{-1}{\text{D}}_{\varrho }b_{\varrho }\left(t\right). \end{array}$$

The evolution over time of the ${\hat{\text{E}}}_{\varrho }\left(t\right)$, estimated at the center *c* of each ϱ-type clique, describes a loop trajectory in the 2D plane. Under the assumption of locally plane and homogeneous wave, *E*_⊥_(*t*) ≈ 0 within the clique, so that ${\hat{\text{E}}}_{\varrho }\left(t\right)$ should have a dominant direction and the loop should lie on a straight line along the propagation direction ${\vec{\text{u}}}_{p}$ (Deno et al., 2017). On the other hand, if the wave is not plane, ${\hat{\text{E}}}_{\varrho }\left(t\right)$ will describe a bidimensional loop and propagation cannot be characterized by the projection on a single direction.

### 2.5. Electric Field Estimates at Each Clique

If *N* × *N* is the size of the MEA, there will be (*N* − 1) × (*N* − 1) = *N*^2^ − 2*N* + 1 estimates of $\hat{\text{E}}\left(t\right)$, in the case of square cliques, and four times this value in the case of triangular configurations. Using Equation (17) and the definitions in Equations (4, 16), the two components Ê_*x*_(*t*) and Ê_*y*_(*t*) of the electric field can be expressed as linear combinations of the two b-EGMs along *x*- and *y*- directions, respectively:

(18) $$\begin{array}{l} {\hat{E}}_{□}\left(t\right):\{\begin{array}{c} {Ê}_{x}\left(t\right)=\frac{1}{2d}\left(b_{12}\left(t\right)+b_{34}\left(t\right)\right)=-\frac{1}{v}\sin\theta \phi^{\prime }\left(t-t_{c}\right)\\ {Ê}_{y}\left(t\right)=\frac{1}{2d}\left(b_{13}\left(t\right)+b_{24}\left(t\right)\right)=-\frac{1}{v}\cos\theta \phi^{\prime }\left(t-t_{c}\right) \end{array} \end{array}$$

(19) $$\begin{array}{l} {\hat{E}}_{△,1}:\{\begin{array}{c} {Ê}_{x}\left(t\right)=\frac{1}{d}b_{12}\left(t\right)≅-\frac{1}{v}\sin\theta \phi^{\prime }\left(t-t_{c}+\frac{d}{2v}\cos\theta \right)\\ {Ê}_{y}\left(t\right)=\frac{1}{d}b_{13}\left(t\right)≅-\frac{1}{v}\cos\theta \;\phi^{\prime }\left(t-t_{c}+\frac{d}{2v}\sin\theta \right) \end{array} \end{array}$$

(20) $$\begin{array}{l} {\hat{E}}_{△,2}:\{\begin{array}{c} {Ê}_{x}\left(t\right)=\frac{1}{d}b_{12}\left(t\right)≅-\frac{1}{v}\sin\theta \phi^{\prime }\left(t-t_{c}+\frac{d}{2v}\cos\theta \right)\\ {Ê}_{y}\left(t\right)=\frac{1}{d}b_{24}\left(t\right)≅-\frac{1}{v}\cos\theta \phi^{\prime }\left(t-t_{c}-\frac{d}{2v}\sin\theta \right) \end{array} \end{array}$$

(21) $$\begin{array}{l} {\hat{E}}_{△,3}:\{\begin{array}{c} {Ê}_{x}\left(t\right)=\frac{1}{d}b_{34}\left(t\right)≅-\frac{1}{v}\sin\theta \phi^{\prime }\left(t-t_{c}-\frac{d}{2v}\cos\theta \right)\\ {Ê}_{y}\left(t\right)=\frac{1}{d}b_{13}\left(t\right)≅-\frac{1}{v}\cos\theta \phi^{\prime }\left(t-t_{c}+\frac{d}{2v}\sin\theta \right) \end{array} \end{array}$$

(22) $$\begin{array}{l} {\hat{E}}_{△,4}:\{\begin{array}{c} {Ê}_{x}\left(t\right)=\frac{1}{d}b_{34}\left(t\right)≅-\frac{1}{v}\sin\theta \phi^{\prime }\left(t-t_{c}-\frac{d}{2v}\cos\theta \right)\\ {Ê}_{y}\left(t\right)=\frac{1}{d}b_{24}\left(t\right)≅-\frac{1}{v}\cos\theta \phi^{\prime }\left(t-t_{c}-\frac{d}{2v}\sin\theta \right), \end{array} \end{array}$$

where the approximated terms make use of Equations (7–10).

Equations (18–22) show that the two electric field components within each clique have a different amplitude, depending on the propagation angle θ. In addition, for triangular cliques, there is also a delay between both components, which is also a function of the propagation angle θ and the conduction velocity magnitude *v*. Such delay represents the difference in the wavefront arrival time to the middle point of each bipole.

### 2.6. Time Alignment of b-EGMs

In the formulation of Equation (15), no delay is considered among the b-EGMs. However, when estimating the electric fields making use of measured b-EGMs, the different bipolar signals are activated at their corresponding wavefront arrival times, thus having a delay among them, as expressed in Equations (7–12).

For the square cliques, from Equation (18) we found that there are no delays between Ê_*x*_(*t*) and Ê_*y*_(*t*), for plane wave propagation and to that level of approximation. Consequently, the ${\hat{\text{E}}}_{□}\left(t\right)$ loop should lie on a one-dimensional trajectory. However, each component Ê_*x*_(*t*) and Ê_*y*_(*t*) in Equation (18) is the summation of two terms which have a delay between them: Equations (7–10), respectively. It is well known that the summation of delayed components result in smoothed estimates, (Sörnmo and Laguna, 2005), which can lead to underestimate ${\hat{\text{E}}}_{□}\left(t\right)$ at the peak of the signal. Therefore, the first-order Taylor series approximation used in this work to arrive to Equation (18) no longer applies. This phenomenon can be observed in the second graph of row in **Figure 5D** within the Results section, where the E-field estimate is underestimated at the peak of the signal.

For the triangular cliques, Equations (19–22) show that, in general, there is a delay between Ê_*x*_(*t*) and Ê_*y*_(*t*) components. This misalignment will be reflected at the ${\hat{\text{E}}}_{△}\left(t\right)$ loop trajectory, which thus will lie in a bidimensional plane rather than on the unidimensional line in the wave propagation direction. To avoid these phenomena and to have well synchronized Ê_*x*_(*t*) and Ê_*y*_(*t*), in this study we propose a modified version of the least squares estimator of ***E***_ϱ_(*t*), where the b-EMGs in the clique are previously time-aligned. For that purpose, both in square and in triangular configurations, the time delay τ_*mn*_ between each independent bipolar signal *b*_*mn*_(*t*), *mn* ∈ {12, 34, 13, 24} and the b-EGM with the highest amplitude within the clique *b*_*max*_(*t*) was estimated by maximizing their cross-correlation:

(23) $$\begin{array}{l} \tau_{mn}=\arg\underset{\tau }{\max}\int_{-\infty }^{\infty }b_{mn}\left(t-\tau \right)b_{max}\left(t\right)dt. \end{array}$$

Each b-EGM *b*_*mn*_(*t*) was then advanced by τ_*mn*_ so that it is aligned with *b*_*max*_(*t*). **Figure 5** shows examples of E-field estimated with and without previous alignment.

### 2.7. Estimation of Propagation Angle

OP-EGM approach estimates propagation direction by finding the angle $\hat{\theta }$ which maximizes the cross-correlation between the first time derivative of the unipolar voltage signal $\phi^{\prime }\left(t-t_{c}\right)$, associated to the clique *c*, and the projection of the electric field in that direction Ê_*p*_(*t*). The unipolar voltage signal $\phi^{\prime }\left(t-t_{c}\right)$ at a given clique corresponds to a locally estimated u-EGM, $u_{c}^{\prime }\left(t\right)$, eventually delayed by τ_*c*_: $\phi^{\prime }\left(t-t_{c}\right)=u_{c}^{\prime }\left(t-\tau_{c}\right)$. In the latter equation, $u_{c}^{\prime }\left(t\right)$ must be estimated for each clique as explained later. In the coordinate system depicted in Figure 1D, the E-field ***E***(*t*) is expressed as $\text{E}\left(t\right)={\text{E}}_{x}\left(t\right){\vec{\text{u}}}_{x}+{\text{E}}_{y}\left(t\right){\vec{\text{u}}}_{y}$. Therefore, the projection of ***E***(*t*) along the propagation direction is given by:

(24) $$\begin{array}{l} E_{p}\left(t\right)=E\left(t\right){\vec{\text{u}}}_{p}\\ \;=E_{x}\left(t\right)\sin\left(\theta \right)+E_{y}\left(t\right)\cos\left(\theta \right). \end{array}$$

The estimated conduction angle $\hat{\theta }$ and the possible delay ${\hat{\tau }}_{c}$ will be found by replacing the E-field **E**(*t*) with its estimate according to Equations (18–22) and solving:

(25) $$\begin{array}{l} {}[\hat{\theta },{\hat{\tau }}_{c}]=\underset{\theta ,\tau_{c}}{\text{arg max}}[\sin(\theta )\int_{-\infty }^{\infty }u_{c}^{\prime }(t-\tau_{c}){\hat{E}}_{x}(t)dt\\ \;+\cos(\theta )\int_{-\infty }^{\infty }u_{c}^{\prime }(t-\tau_{c}){\hat{E}}_{y}(t)dt]. \end{array}$$

If $\hat{\tau_{c}}$ is the estimated delay τ_*c*_ maximizing the previous cross-correlation, the propagation angle estimate is:

(26) $$\begin{array}{l} \hat{\theta }=\arctan\frac{\int_{-\infty }^{\infty }u_{c}^{\prime }\left(t-{\hat{\tau }}_{c}\right){Ê}_{x}\left(t\right)dt}{\int_{-\infty }^{\infty }u_{c}^{\prime }\left(t-{\hat{\tau }}_{c}\right){Ê}_{y}\left(t\right)dt}. \end{array}$$

For square cliques, considering the estimates of the E-field components for plane wave propagation in Equation (18), the expressions in Equation (3) and the fact that $u_{c}^{\prime }\left(t-\tau_{c}\right)=\phi^{\prime }\left(t-t_{c}\right)$, we find from Equation (26) that $\hat{\theta }=\theta$, i.e., $\hat{\theta }$ is an unbiased estimate.

Using a similar procedure for triangular cliques, as clique 1 in Figure 3B, considering the fact that $u_{c}^{\prime }\left(t\right)$ and $u_{c}^{\prime \prime }\left(t\right)$ are orthogonal and using the approximation in Equation (19), we obtain that the propagation angle estimate when no previous time alignment of b-EGMs is performed can be expressed as:

(27) $$\begin{array}{l} \hat{\theta }=\arctan\frac{-\frac{1}{v}\sin\theta \int_{-\infty }^{\infty }\phi^{\prime }\left(t-t_{c}+\tau_{c}-{\hat{\tau }}_{c}\right)\;\phi^{\prime }\left(t-t_{c}+\frac{d}{2v}\cos\theta \right)dt}{-\frac{1}{v}\cos\theta \int_{-\infty }^{\infty }\phi^{\prime }\left(t-t_{c}+\tau_{c}-{\hat{\tau }}_{c}\right)\;\phi^{\prime }\left(t-t_{c}+\frac{d}{2v}\sin\theta \right)dt}\neq \theta . \end{array}$$

This result points out that the estimate of θ is biased in general for triangular cliques. Nevertheless, if the electric field components Ê_*x*_(*t*) and Ê_*y*_(*t*) are estimated from properly aligned bipolar signals, then we obtain $\hat{\theta }=\theta$. For cliques corresponding to triangles 2, 3 and 4, similar expressions are obtained with only changes in the signs of the sin(θ) and cos(θ) terms.

In the reference omnipolar method introduced by Deno et al. (2017), it is not specified which of the unipolar signals measured in a clique is used as the $u_{c}^{\prime }\left(t\right)$ in Equation (25). In this work, when reproducing the method proposed in literature, we take the signal from lower left corner electrode when using square cliques, so that *u*_*c*_(*t*) = *u*_1_(*t*). For triangular cliques, the signal from the electrode corresponding to the vertex of the right angle is chosen, so that *u*_*c*_(*t*) = *u*_*k*_(*t*), *k* = {1, 2, 3, 4} for each of the four triangles indicated with the same *k*-th index, respectively, in Figure 3B.

Alternatively, we propose in this work a more robust approach, where the estimation of $u_{c}^{\prime }\left(t\right)$ to be included in Equation (25) involves all the u-EGMs within the clique. For the case of square clique:

1. each of the four u-EGMs is aligned with respect to the one with the highest amplitude, denoted as *u*_*max*_(*t*), by computing the maximum cross-correlation between them. In this way, the four aligned u-EGMs *u*_*k*_(*t* − τ_*k*_), *k* ∈ {1, 2, 3, 4}, are obtained;
2. the average signal ū(*t*) is calculated by averaging the four aligned u-EGMs *u*_*k*_(*t* − τ_*k*_);
3. the time derivative of ū(*t*) is computed and used as estimate of $u_{c}^{\prime }\left(t\right)$ in Equation (25), $u_{c}^{\prime }\left(t\right)=\partial ū\left(t\right)/\partial t$.

For triangular cliques, steps 1. and 2. are performed by just considering the three u-EGMs at the electrodes forming the clique. Step 3. is the same as for square cliques.

### 2.8. Estimation of Conduction Velocity

According to the omnipolar method, the conduction velocity *v* in each clique is estimated by comparing the amplitudes of $\phi^{\prime }\left(t-t_{c}\right)$ and Ê_*p*_(*t*), as they are expected to be proportional under the assumption of locally plane wave: ${Ê}_{p}\left(t\right)=-\phi^{\prime }\left(t-t_{c}\right)/v$, if the b-EGMs within the clique are aligned (see Equations 18–22). The conduction velocity *v* can be estimated by computing the ratio of the peak-to-peak (pp) amplitudes of $\phi^{\prime }\left(t-t_{c}\right)$ and Ê_*p*_(*t*), as proposed by Deno et al. (2017):

(28) $$\begin{array}{l} \hat{v}=\frac{{\left[\phi^{\prime }\left(t-t_{c}\right)\right]}_{\text{pp}}}{{\left[{Ê}_{p}\left(t\right)\right]}_{\text{pp}}}. \end{array}$$

In order to avoid the large sensitivity to noise that be expected from an estimation based on peak-to-peak amplitudes, we propose here a more robust estimate of *v* as the ratio of the standard deviations (SD) of $\phi^{\prime }\left(t-t_{c}\right)$ and Ê_*p*_(*t*):

(29) $$\begin{array}{l} \hat{v}=\frac{{\left[\phi^{\prime }\left(t-t_{c}\right)\right]}_{\text{SD}}}{{\left[{Ê}_{p}\left(t\right)\right]}_{\text{SD}}}. \end{array}$$

For square cliques, by using Equation (18) in Equation (24) and if the propagation direction is well estimated ($\hat{\theta }=\theta$), simple calculations lead to the expected:

(30) $$\begin{array}{l} {Ê}_{p}\left(t\right)=-\frac{1}{v}\phi^{\prime }\left(t-t_{c}\right). \end{array}$$

For triangular cliques (e.g., clique 1), using the approximations with delays in Equation (19) for Ê_*x*_(*t*) and Ê_*y*_(*t*), and assuming that the propagation direction is well estimated ($\hat{\theta }=\theta$), we have:

(31) $$\begin{array}{l} {\hat{E}}_{p}(t)\text{​}≅\text{​}-\frac{1}{v}(\sin^{2}\theta \;\phi^{\prime }\left(t-t_{c}+\frac{d}{2v}\cos\theta \right)\\ +{\text{cos}}^{2}\theta \;\phi^{\prime }\left(t-t_{c}+\frac{d}{2v}\sin\theta \right)). \end{array}$$

This equation shows that the misalignment of $\hat{E_{y}}\left(t\right)$ and $\hat{E_{x}}\left(t\right)$ components in triangular cliques has an effect in the conduction velocity estimate, even if propagation direction is estimated without error. Nevertheless, if the b-EGMs are time aligned as proposed in this work (i.e., if there are not delays between $\hat{E_{y}}\left(t\right)$ and $\hat{E_{x}}\left(t\right)$), the approximation in Equation (31) reduces to an expression analogous to Equation (30), allowing a proper estimation of the conduction velocity.

### 2.9. Omnipolar Signals for Voltage Maps

In this work, OP-EGM signals φ_*i, j*, (*q*)_(*t*), where *q* ∈ {1, 2, 3, 4} refers to each of the four possible triangles when using triangular clique configuration, were defined by projecting $\hat{\text{E}}$(*t*) within the corresponding clique onto the following directions:

- The direction ${\vec{\text{u}}}_{me}$ that maximizes the excursion of the $\hat{\text{E}}\left(t\right)$ loop within the depolarization interval. In that case, the OP-EGM signal $\varphi_{i,j,\left(q\right)}^{me}\left(t\right)$ along the loop maximal excursion (*me*) direction was derived as: $\varphi_{i,j,\left(q\right)}^{me}\left(t\right)=d\cdot \;\hat{\text{E}}\left(t\right)\cdot {\vec{\text{u}}}_{me}$. It should be noted that the peak-to-peak amplitude of $\varphi_{i,j,\left(q\right)}^{me}\left(t\right)$ is equivalent to the peak-to-peak amplitude *E*_*max*_ of the electric field, proposed by Deno et al. (2017), multiplied by the inter-electrode distance within the clique.
- The principal direction ${\vec{\text{u}}}_{\nu }$ obtained by the principal component analysis (*pca*) of the loop described by $\hat{\text{E}}$(*t*) within the depolarization interval. The resulting OP-EGM signal $\varphi_{i,j,\left(q\right)}^{pca}\left(t\right)$ was derived as: $\varphi_{i,j,\left(q\right)}^{pca}\left(t\right)=d\cdot \;\hat{\text{E}}\left(t\right)\cdot {\vec{\text{u}}}_{\nu }$. In this approach, we also obtained the voltage projected in the orthogonal direction ${\vec{\text{u}}}_{\nu }^{⊥}$ (corresponding to the second principal component): $\varphi_{i,j,\left(q\right)}^{pca⊥}\left(t\right)=d\cdot \;\hat{\text{E}}\left(t\right)\cdot {\vec{\text{u}}}_{\nu }^{⊥}$.

Let us consider $\varphi_{i,j,\left(q\right)}^{me}\left(t\right)$ under the plane wave assumption. For square cliques, if maximal excursion occurs in the propagation direction and conduction angle is well estimated, we will have:

(32) $$\begin{array}{l} \varphi_{i,j}^{me}\left(t\right)=-\frac{d}{v}\phi^{\prime }\left(t-t_{c}\right). \end{array}$$

It should be noted that there are no second-order terms in Equation (32), unlike the expressions of b-EGMs (see Equations 7–12). Again, alignment prevents attenuation at the $\hat{\text{E}}\left(t\right)$ peaks and consequently yields a better estimate. For triangular cliques under the same assumptions (let us consider clique 1, for instance), there are indeed second order terms in $\varphi_{i,j,1}^{me}\left(t\right)$, affecting omnipolar signal amplitude. Nevertheless, expressing Ê_*y*_(*t*) and Ê_*x*_(*t*) components with the delay approximation in Equation (19) and time aligning them, we obtain the aligned version $\varphi_{i,j,1}^{me,a}\left(t\right)$:

(33) $$\begin{array}{l} \varphi_{i,j,1}^{me,a}\left(t\right)=-\frac{d}{v}\phi^{\prime }\left(t-t_{c}+\frac{d}{2v}\cos\theta \right), \end{array}$$

whose amplitude is properly estimated. Examples of the omnipolar EGM signals here introduced are observed in **Figure 6**, for both clique configurations and the three MEA orientations, with and without the previous alignment of b-EGMs.

### 2.10. Voltage and Conduction Velocity Maps

For each OP-EGM signal introduced in this work $\varphi_{i,j,\left(q\right)}^{s}\left(t\right)$, *s* ∈ {*me, pca, pca*⊥}, we computed the peak-to-peak amplitude $V_{i,j,\left(q\right)}^{o\text{-}s}$, which was used to build voltage maps. In the *pca* approach, we also created maps based on the root sum square of the voltage in the first and second principal components, $V_{i,j,\left(q\right)}^{o\text{-}pca-r}$:

(34) $$\begin{array}{l} V_{i,j,\left(q\right)}^{o\text{-}s}=\underset{t}{\max}\left\{\varphi_{i,j,\left(q\right)}^{s}\left(t\right)\right\}-\underset{t}{\min}\left\{\varphi_{i,j,\left(q\right)}^{s}\left(t\right)\right\},\\ \;i\in \left\{1,..,N-1\right\},j\in \left\{1,..,N-1\right\},s\in \left\{me,pca,pca⊥\right\},\\ V_{i,j,\left(q\right)}^{o\text{-}pca\text{-}r}=\sqrt{{\left(V_{i,j,\left(q\right)}^{o\text{-}pca}\right)}^{2}+{\left(V_{i,j,\left(q\right)}^{o\text{-}pca⊥}\right)}^{2}},\\ \;i\in \left\{1,..,N-1\right\},j\in \left\{1,..,N-1\right\}. \end{array}$$

We also built bipolar maps based on the peak-to-peak amplitudes $V_{i,j}^{b\text{-}x}$ and $V_{i,j}^{b\text{-}y}$ of the b-EGMs in each of the two catheter directions (i.e., $b_{i,j}^{x}\left(t\right)$ and $b_{i,j}^{y}\left(t\right)$). The root sum square (*r*) of both values, as well as their maximum (*m*) were also considered so as to have a performance reference based on bipolar signals:

(35) $$\begin{array}{l} V_{i,j}^{b\text{-}r}=\sqrt{{\left(V_{i,j}^{b\text{-}x}\right)}^{2}+{\left(V_{i,j}^{b\text{-}y}\right)}^{2}};\;V_{i,j}^{b\text{-}m}=\max\left\{V_{i,j}^{b\text{-}x},V_{i,j}^{b\text{-}y}\right\},\\ \;i\in \left\{1,..,N-1\right\},j\in \left\{1,..,N-1\right\}. \end{array}$$

All the voltage maps were computed for each orientation of the MEA, and by using both clique configurations analyzed in this study. Omnipolar voltage maps were also performed from aligned b-EGMs. We obtained color-coded maps where a pixel corresponds to a clique (or to an electrode pair, in case of bipolar maps).

Regarding conduction velocity ($\text{v}=v{\vec{\text{u}}}_{v}$), we created maps for each catheter orientation, each clique configuration, with and without alignment of b-EGMs. More specifically, each map consists of color-coded *v*-values and arrows representing angular direction θ of the estimated velocity vectors ***v*** in each clique. Plotted *v*-values were estimated by using both OP-EGM approach of the Equation (28) proposed by Deno et al. (2017) and the modified version MOP-EGM introduced in this work in Equation (29).

It should be noted that maps performed with square cliques present the same resolution as bipolar maps, both providing *N*^2^ − 2*N* + 1 pixels when processing the whole MEA. On the other hand, since triangular cliques result in a total of 4 × (*N*^2^ − 2*N* + 1) measurement configurations (and consequently, pixels) within the MEA, maps performed with this configuration present higher spatial resolution.

### 2.11. Voltage and Conduction Velocity Maps From Noisy Signals

In order to assess the sensitivity to noise of voltage and conduction velocity maps, simulated b-EGMs were corrupted with noise obtained from real b-EGMs recorded at Hospital Santa Marta, Lisbon. Six hundred different noise segments were extracted from b-EGMs recorded with a PentaRay^®^ catheter (*Biosense-Webster, Inc., Diamond Bar, CA, USA*) in 27 different mapping points at intervals with no recorded EGMs. All noise segments were normalized in order to guarantee the same power level, coinciding with the observed average power. One hundred different realizations of this recorded noise were randomly added to each of the simulated b-EGMs $b_{i,j}^{x}\left(t\right)$ and $b_{i,j}^{y}\left(t\right)$ along *x* and *y* directions of the MEA. As a result, we generated one hundred noisy bipolar signals along each of the two directions, $b_{i,j}^{x,n}\left(t\right)$ and $b_{i,j}^{y,n}\left(t\right)$. For each realization *n*, the different modalities of voltage and conduction velocity maps were computed, obtaining 100 different maps from noisy b-EGMs. These mapping strategies were tested regarding fibrosis detection, as well as voltage map reproducibility. The previous procedure was repeated for six different noise levels, scaling the noise realizations so that they had standard deviations σ_*n*_ ∈ {3, 6, 14, 28, 42, 55} μ*V*. The standard deviation of the recorded noise ranged from 1.2 μ*V* to 11 μ*V*, with an average value of 2.4 μ*V*, lower than those reported in Unger et al. (2019). We simulated noise levels ranging up to 55 μ*V* which approximately corresponds to the 95th percentile of the bipolar noise levels reported in Unger et al. (2019), in order to test the performance of the mapping strategies at challenging situations. An example of noisy b-EGM $b_{14,2}^{y}\left(t\right)$ with noise level σ_*n*_ = 14 μ*V* is shown in Supplementary Figure 2C.

### 2.12. Maps Performance Evaluation for Fibrosis Detection

In order to quantitatively evaluate the ability of the different mapping strategies to identify the fibrotic area (i.e., to discriminate pixels within the fibrotic patch from those in the normal tissue), receiver operating characteristic (ROC) curves were used. Two ground-truth masks of fibrosis were created for bipolar and omnipolar maps, having the resolution of square and triangular cliques and indicating whether a clique lay within the fibrotic or the normal tissue. A 14 × 14 ground-truth mask was used to evaluate bipolar and omnipolar maps with square cliques. A value of 1 was assigned if the four electrodes defining the square clique lay in the fibrotic area, and a value of 0 was assigned if all of them lay in the non-fibrotic area. Cliques with some electrodes inside and some outside the fibrotic patch were not considered in the evaluation. Similarly, a 28 × 28 ground-truth mask was used to assess omnipolar maps performed with triangular cliques, considering if the three electrodes defining the triangular clique lay in fibrotic or non-fibrotic tissue.

**Figure 4:**
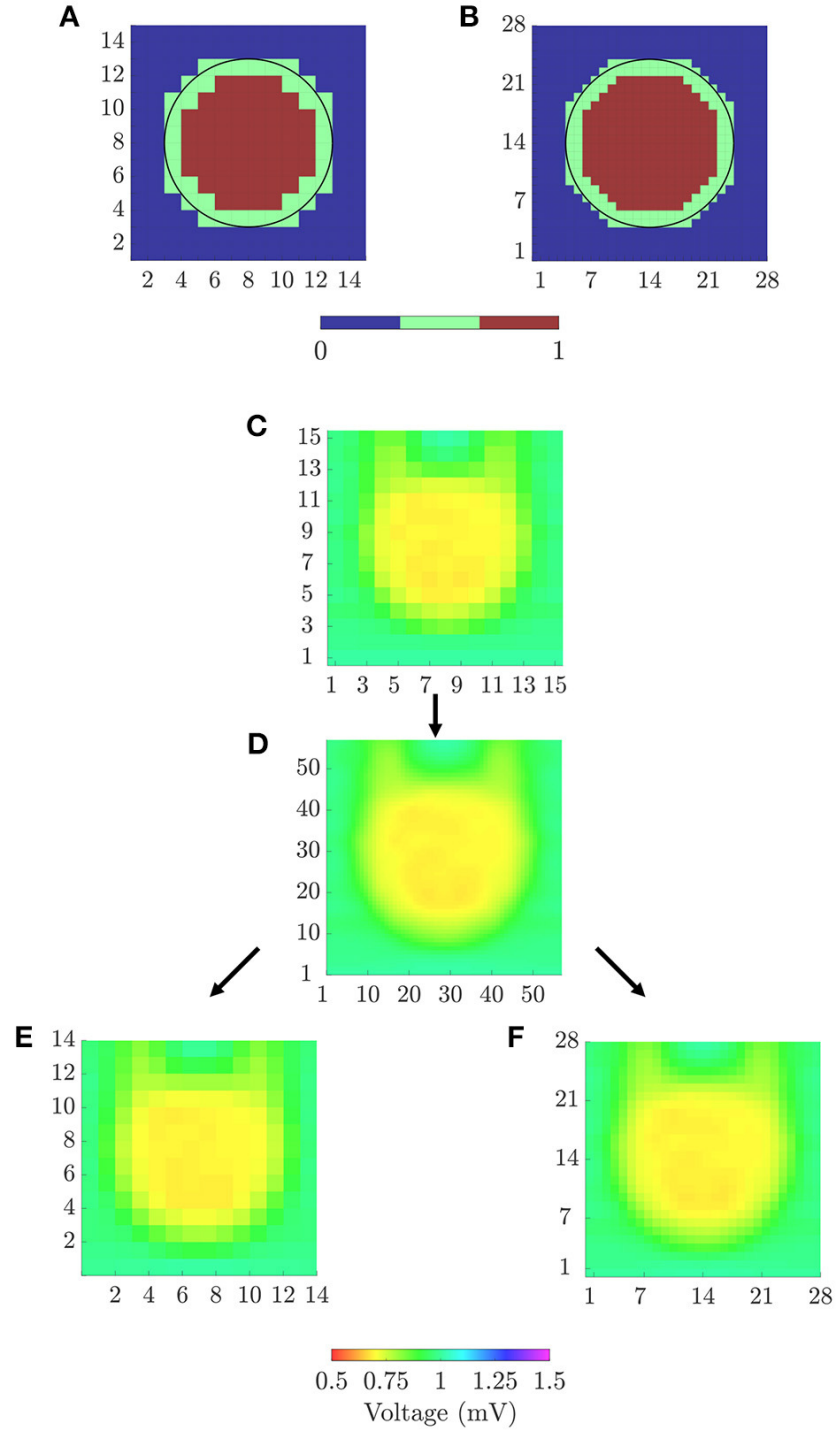
**(A)** 14 × 14 and **(B)** 28 × 28 ground-truth masks for fibrosis detection, for square and triangular cliques, respectively. **(C)** 15 × 15 peak-to-peak amplitudes map from u-EGMs and **(D)** 57 × 57 map resulting from bi-cubic interpolation of **(C)**. 14 × 14 **(E)** and 57 × 57 **(F)** amplitude maps for evaluating voltage reproducibility of bipolar and omnipolar maps.

Both ground-truth masks used in this study are illustrated in Figures 4A,B. For each voltage and velocity map, ROC curves relating sensitivity and specificity were obtained by varying the threshold for fibrosis identification (Metz, 1978). In this analysis, *true positive* denotes the number of cliques correctly identified as fibrotic, *false negative* represents the number of missed cliques in the fibrotic area, *true negative* stands for the number of cliques correctly identified as non-fibrotic and *false positive* is the number of cliques incorrectly detected as fibrotic. The accuracy (*ACC*) was defined as the number of cliques correctly identified as fibrotic or non-fibrotic divided by the total number of cliques. The maximum *ACC* was used as a measure of the overall fibrosis detection ability of a given map. In addition, the sensitivity and specificity with the threshold achieving the maximum *ACC* were also computed.

### 2.13. Maps Performance Evaluation for Reproducibility of the Reference Voltage

The ability of the voltage mapping strategies in reproducing the spatial distribution of reference maps was also evaluated. We created peak-to-peak amplitude maps of u-EGMs, and resampled them to match the spatial resolutions of bipolar and omnipolar pixel maps (with both square and triangular cliques). To quantify the agreement of each voltage map with the substrate characterization given by the reference map, we used both Pearson's ρ_*p*_ and Spearman's rank ρ_*s*_ correlation coefficients. A *p* ≤ 0.05 was required to have statistical significance. Reference voltage maps were obtained by following two sequential steps:

1. A bicubic interpolation of the *N* × *N* peak-to-peak amplitude map of u-EGMs *u*_*i, j*_(*t*), (*i, j*) ∈ {1, …, *N*}, was performed placing three interpolated values between any two adjacent electrodes, along both MEA directions, thus increasing the total number of pixels to (*N* + 3 · (*N* − 1)) × (*N* + 3 · (*N* − 1)). The original and interpolated maps for *N* = 15 are shown in Figures 4C,D, respectively, when MEA is oriented with Ψ = 0°.
2. The interpolated pixel map was later resampled so as to obtain (*N* − 1) × (*N* − 1) and 2·(*N* − 1) × 2·(*N* − 1) reference maps, matching the dimensions of bipolar and omnipolar maps with square cliques, and omnipolar maps with triangular cliques, respectively. Resulting resampled pixel maps from *N* = 15, for the case when Ψ = 0°, are illustrated in Figures 4E,F.

### 2.14. Error Evaluation for Noisy Maps

Additionally, in order to evaluate the effect of noise on voltage and velocity maps, we computed the root mean square error (RMSE) between each noisy map and the respective noise-free map. In case of voltage maps, we calculated:

(36) $$\begin{array}{l} \text{RMSE}\left(n\right)=\sqrt{\frac{1}{{\left(N-1\right)}^{2}}\overset{N-1}{\underset{i=1}{\sum }}\overset{N-1}{\underset{j=1}{\sum }}{\left(V_{i,j}^{s,n}-V_{i,j}^{s}\right)}^{2}}, \end{array}$$

where *s* ∈ {*b*-*m*; *b*-*r*; *o*-*me*; *o*-*me, a*; *o*-*pca*-*r*; *o*-*pca*-*r, a*}. Analogously, for velocity maps, we computed:

(37) $$\begin{array}{l} \text{RMSE}\left(n\right)=\sqrt{\frac{1}{{\left(N-1\right)}^{2}}\overset{N-1}{\underset{i=1}{\sum }}\overset{N-1}{\underset{j=1}{\sum }}{\left(CV_{i,j}^{s,n}-CV_{i,j}^{s}\right)}^{2}}, \end{array}$$

with *s* ∈ {*o*; *o*-*m*; *o*-*m, a*}.

We also studied the effect of noise on the estimation of the propagation direction with both the original θ^*o*^ and the modified θ^*o*-*m*^ omnipolar method. Angle differences, ϵ_θ_, between propagation direction maps obtained from noisy realizations and those obtained from noise-free signals (considered as the reference) were computed for each omnipolar approach and noise level. The mean and standard deviation of the angle differences in each map were obtained and averaged for all noise realizations.

All performance measurements were reported jointly for the three MEA orientations considered in this study, thus assuming that the relative angle of the propagation direction with respect to the catheter is not known a priori.

## 3. Results

**Figure 5:**
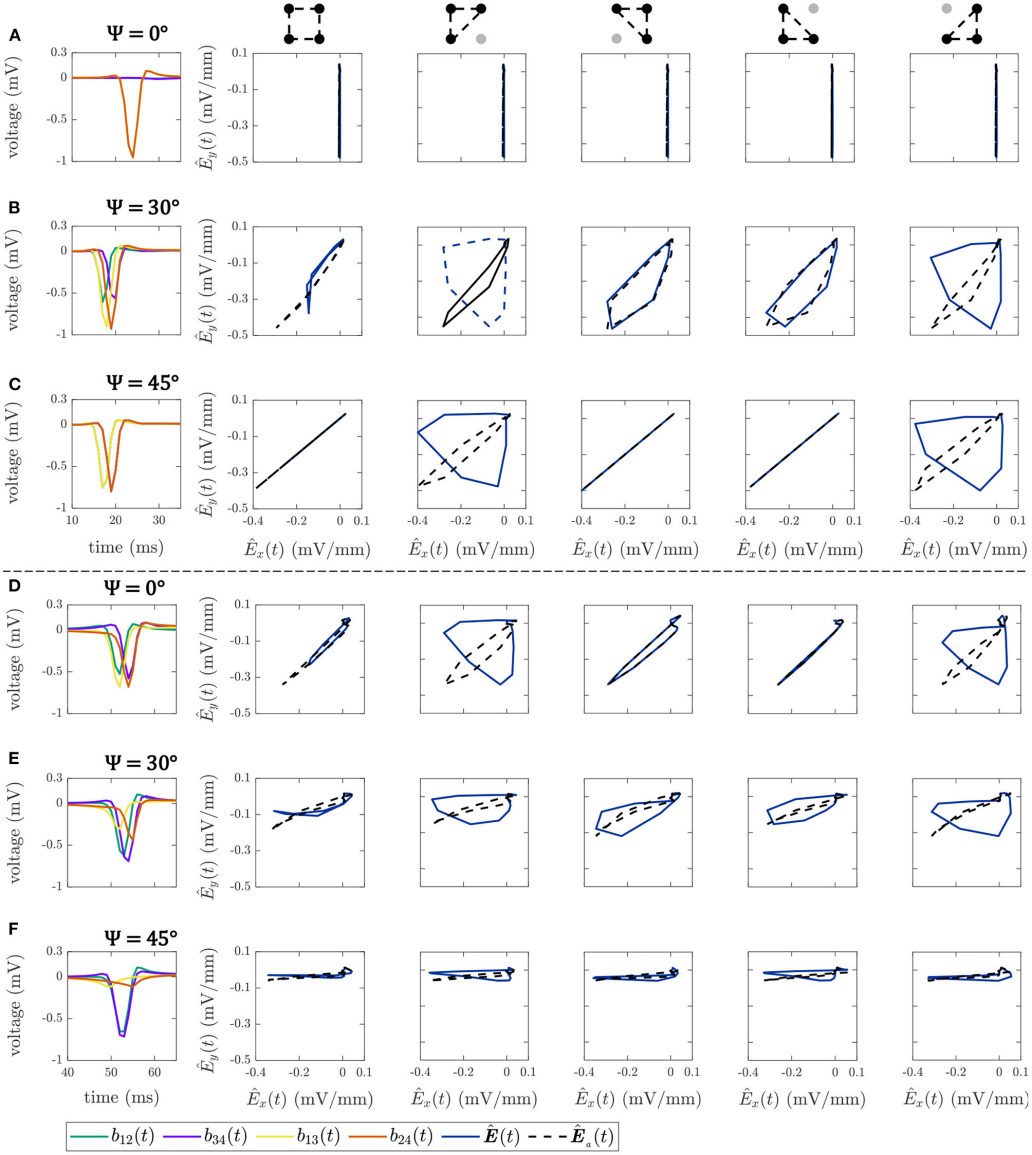
Bipolar EGMs (first column) and E-field loops (remaining columns) obtained with (dashed line) and without (solid line) the alignment of b-EGMs, in square and triangular cliques. All the cliques represented are from non-fibrotic areas outside the fibrotic patch. Rows **(A–C)**: cliques with plane wavefront, when Ψ = 0°, Ψ = 30° and Ψ = 45°, respectively. Rows **(D–F)**: cliques with curved wavefront, when Ψ = 0°, Ψ = 30° and Ψ = 45°, respectively.

Figure 5 shows some illustrative examples of the electric field loops estimated in both square and triangular configurations, together with their respective b-EGMs, when MEA was oriented with Ψ = 0°, Ψ = 30° and Ψ = 45°, at different (*i, j*) electrode positions. All the E-field loops were estimated with and without previous time alignment of b-EGMs, as proposed in subsection 2.6 (denoted as ***E***_*a*_(*t*) and ***E***(*t*), respectively). In the top three rows, cliques were chosen in a non-fibrotic area with plane wavefront. Rows **A**–**C** correspond to the clique (*i, j*) = (2, 2) for Ψ = 0°, Ψ = 30° and Ψ = 45°, respectively. The estimated local propagation angles with the proposed MOP-EGM method were ${\hat{\theta }}_{2,2}$ = -0.1°, ${\hat{\theta }}_{2,2}$ = 23.5° and ${\hat{\theta }}_{2,2}$ = 45.0°, respectively. Lower panels represent cliques from non-fibrotic areas with curved wavefronts: (*i, j*) = (4, 12) with Ψ = 0° in Figure 5D; (*i, j*) = (7, 13) with Ψ = 30° in Figure 5E; and at (*i, j*) = (8, 13) with Ψ = 45° in Figure 5F. The respective estimated local propagation angles were ${\hat{\theta }}_{4,12}$ = 34.7°, ${\hat{\theta }}_{7,13}$ = 70.3° and ${\hat{\theta }}_{8,13}$ = 85.3°.

The effect of the proposed alignment is evident in both upper and lower panels. In square cliques, the E-field peak value for the aligned versions is clearly higher than for the non-aligned ones. On the other hand, in triangular cliques the alignment of b-EGMs proves to improve the estimation of the propagation direction. These is clearly seen especially in those areas where the local propagation direction differs from the dominant directions of the MEA [see panels **(B)**, **(C)**, **(D)** and **(E)**].

**Figure 6:**
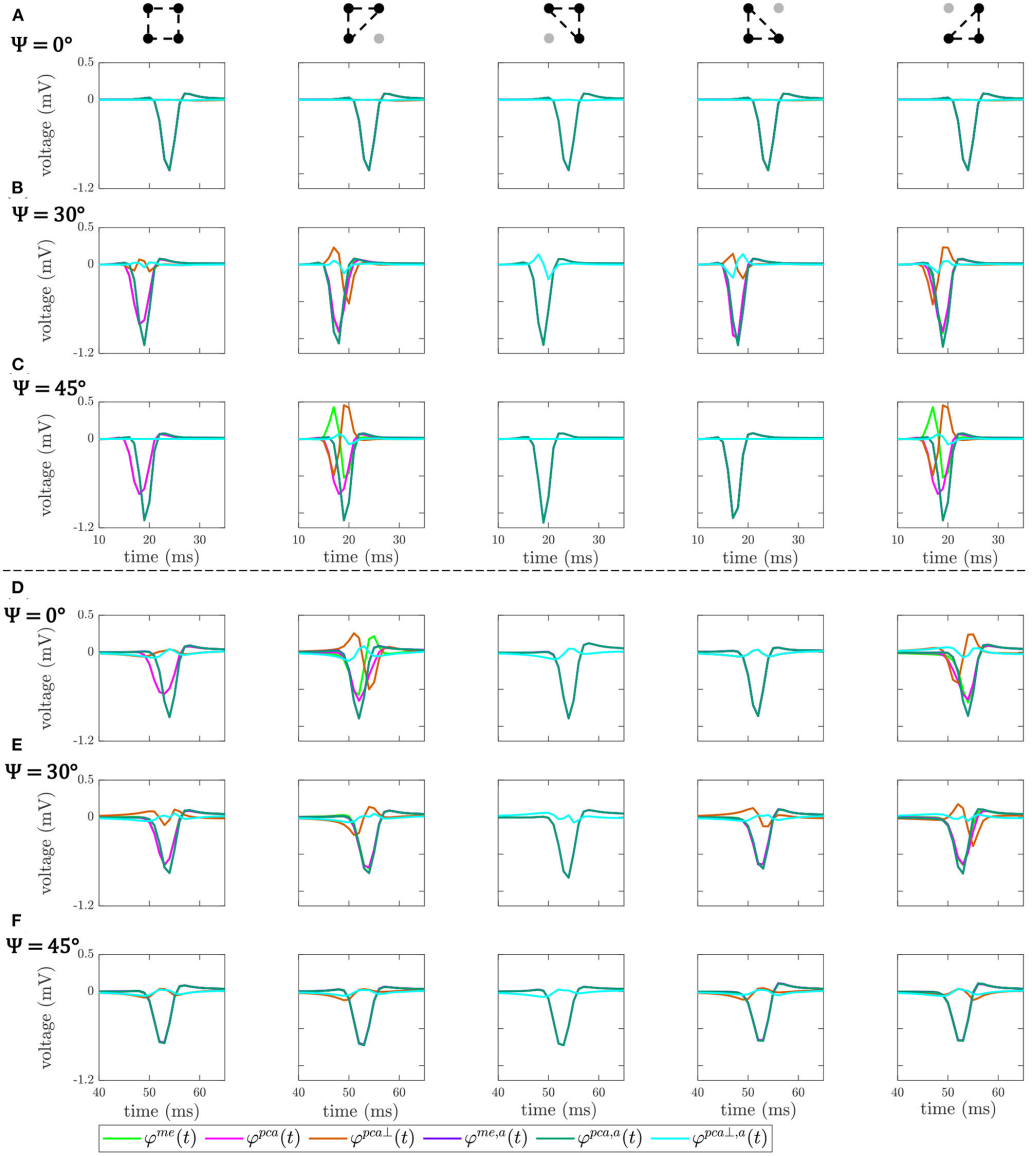
OP-EGM signals $\varphi_{i,j,\left(q\right)}^{s}\left(t\right)$, where *s* ∈ {*me, pca, pca*⊥}, obtained in square and triangular cliques, estimated with (*a*) and without alignment of b-EGMs. All the cliques represented are from non-fibrotic areas outside the fibrotic patch. Rows **(A–C)**: cliques with plane wavefront, when Ψ = 0°, Ψ = 30° and Ψ = 45°, respectively. Rows **(D–F)**: cliques with curved wavefront, when Ψ = 0°, Ψ = 30° and Ψ = 45°, respectively.

Figure 6 shows the OP-EGM signals estimated with the different proposed approaches at the same electrode positions(*i, j*) as in Figure 5: $\varphi_{i,j,\left(q\right)}^{s}\left(t\right)$, where *s* ∈ {*me, pca, pca*⊥}, in square and triangular cliques, with and without alignment of b-EGMs. These results show that the OP-EGM signals produced by the two modalities are quite similar in most of the cases. Square cliques reveal that $\varphi_{i,j}^{me}\left(t\right)$ is equivalent to $\varphi_{i,j}^{pca}\left(t\right)$, both when the b-EGMs are aligned and when they are not. In triangular cliques, this is also true in areas where the propagation direction is parallel to one of the main directions of the MEA.

**Figure 7:**
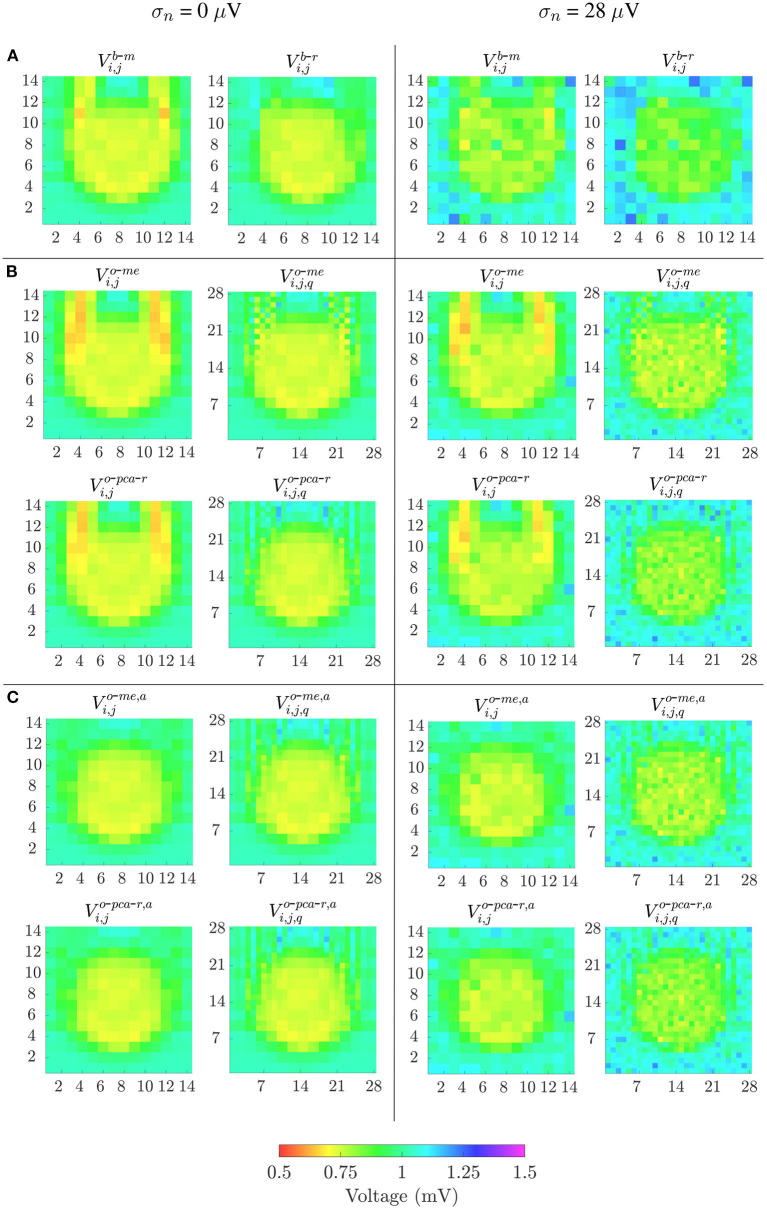
Voltage maps when propagation direction is in the main direction of MEA (Ψ = 0°). Left columns: noise-free bipolar maps (row **A**) and omnipolar maps without (rows in **B**) and with alignment (rows in **C**) of b-EGMs; right columns: same types of maps performed in left columns, but from noisy b-EGMs (noise level with standard deviation σ = 28μ*V*).

The different bipolar and omnipolar voltage mapping strategies are illustrated in Figure 7 for Ψ = 0°, without noise (left columns) and with a noise level of σ_*n*_ = 28μ*V* (right columns). In the latter case, only one of the 100 noisy realizations was shown. The omnipolar maps are presented without and with the alignment of b-EGMs. These results reveal that combined bipolar maps obtained as the root sum square or the maximum voltage of both MEA directions, *V*^*b*-*r*^ and *V*^*b*-*m*^, shown at row **A**, present better fibrosis detection performance than omnipolar maps without alignment, illustrated at upper and lower rows in Figure 7B for the two approaches *me* and *pca*, respectively. However, omnipolar maps with previous time alignment of b-EGMs (upper and lower rows in Figure 7C for the same approaches as in Figure 7B) show results comparable to combined bipolar maps. Omnipolar mapping strategies also present greater correlation with the reference map when time alignment is applied. When b-EGMs are affected by noise, omnipolar voltage maps are more robust than bipolar maps, improving their performance in fibrosis discrimination and correlation with their reference when previous alignment is performed.

**Figure 8:**
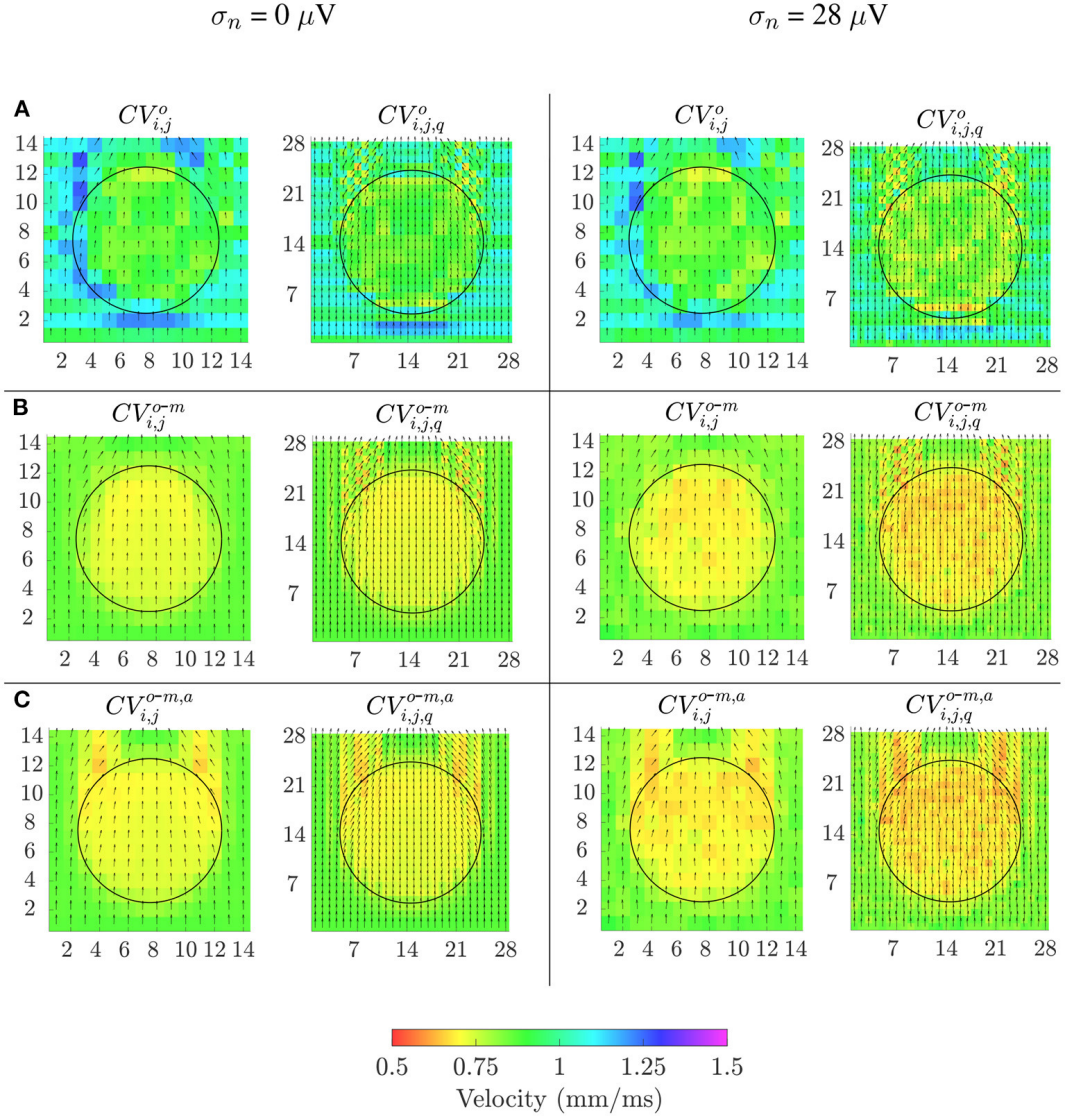
Velocity maps when propagation direction is in the main direction of MEA (Ψ = 0°). Estimated conduction velocities are color-coded and arrows show the estimated propagation direction. Left columns: noise-free velocity maps performed with the reference omnipolar method **(A)** and with the proposed approach (without **(B)** and with alignement **(C)** of b-EGMs). Right columns: same types of maps as in left columns, but performed from noise corrupted b-EGMs for a particular realization and noise level with standard deviation σ = 28μ*V*.

Velocity maps obtained with the reference OP-EGM method (row **A**) and with the proposed approach (without (row **B**) and with (row **C**) alignment of b-EGMs) are presented in Figure 8, at the same MEA orientation and with the same noise level as in Figure 7. These maps reveal better performance of the MOP-EGM method (*CV*^*o*-*m*^ and *CV*^*o*-*m, a*^) than the standard approach (*CV*^*o*^). Moreover, the standard approach tends to overestimate conduction velocity values, when compared with the MOP-EGM method.

AUC, ACC, Pearson ρ_*p*_ and Spearman ρ_*s*_ correlation values of the proposed mapping strategies for noise-free signals are shown in Table 1, together with the threshold value with maximum detection accuracy for each map modality. Note that performance was computed by considering jointly the three MEA orientations with respect to the propagation direction. Bipolar voltage maps *V*^*b*-*m*^ and *V*^*b*-*r*^ identify the fibrotic area with an ACC of 96 and 95%, respectively, whereas the OP-EGM based voltage maps reach a maximum ACC value of 93%, being consistent with results in Figure 7. Regarding correlation of the voltage maps with the reference maps, both omnipolar strategies (*me* and *pca*) achieve values ρ_*p*_ = 0.87 and ρ_*s*_ = 0.87 when square cliques are used and b-EGMs alignment is performed. As a reference, *V*^*b*-*r*^ and *V*^*b*-*m*^ present correlations of ρ_*p*_ = 0.86 and ρ_*p*_ = 0.83, respectively. Regarding CV maps, the MOP-EGM version proposed in this work (with b-EGMs alignment) presents indeed comparable performance in fibrosis detection to combined bipolar voltage maps (e.g., ACC = 96% for *CV*^*o*-*m, a*^ with square cliques), and better performance than both omnipolar voltage maps and than the original OP-EGM approach to estimate conduction velocity (*CV*^*o*^).

**Table 1:** Fibrosis detection performance (voltage threshold, AUC and ACC), Pearson ρ_*p*_ and Spearman's ρ_*s*_ correlations obtained from clean b-EGMs (σ_*n*_ = 0μ*V*), by considering jointly the three MEA orientations with respect to the propagation direction.

**Map type**	**Cliques**	**Threshold (mV)**	***AUC***	***ACC* (%)**	$\rho_{p}^{*}$	$\rho_{s}^{*}$
**Without || with b-EGMs alignment**						
*V* ^*b*-*m*^	-	0.76	0.98	96	0.83	0.82
*V* ^*b*-*r*^	-	0.88	0.97	95	0.86	0.84
*V* ^*o*-*me*^	□	0.75 || 0.90	0.97 || 0.96	92 || 93	0.77 || 0.87	0.79 || 0.87
	△	0.87 || 0.91	0.96 || 0.97	88 || 92	0.83 || 0.86	0.83 || 0.85
*V* ^*o*-*pca*-*r*^	□	0.75 || 0.90	0.97 || 0.96	93 || 93	0.77 || 0.87	0.79 || 0.87
	△	0.93 || 0.90	0.95 || 0.96	89 || 92	0.82 || 0.86	0.82 || 0.85
*CV* ^*o*^	□	0.90	0.77	70	-	-
	△	0.91	0.80	74	-	-
*CV* ^*o*-*m*^	□	0.82 || 0.74	0.95 || 0.98	90 || 96	-	-
	△	0.74 || 0.73	0.92 || 0.97	86 || 94	-	-

^*^All the p < 0.01.

**Figure 9:**
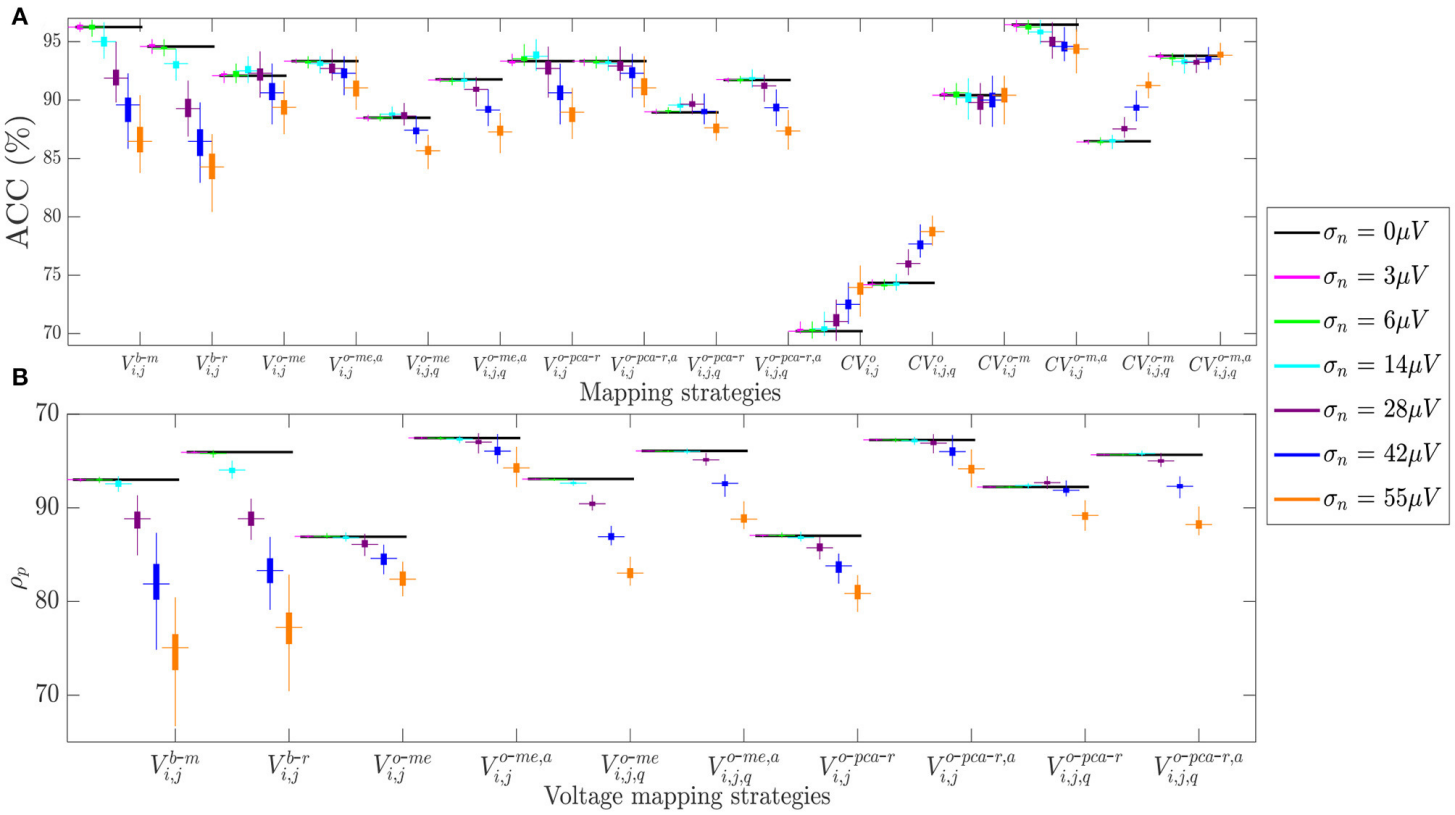
**(A)** Fibrosis detection accuracy (ACC) of the different mapping strategies. **(B)** Pearson's correlation coefficient ρ_*p*_ of the different voltage mapping strategies. For each noise level, the central mark and the bottom and top edges of each box indicate the median, the first and the third quartile, respectively, whereas the noise-free ACC and ρ_*p*_ are depicted as black horizontal lines.

Similar results are observed in Figure 9, which shows how fibrosis detection (in terms of the ACC value, Figure 9A) and voltage map fidelity (in terms of Pearson's correlation coefficient ρ_*p*_, Figure 9B) are affected when b-EGMs are corrupted with different noise levels, jointly considering the three MEA orientations. As observed in panel **A**, omnipolar voltage and CV maps are less affected by noise than bipolar maps for noise levels greater than or equal to σ_*n*_ = 28μ*V*. Note that the proposed time alignment of b-EGMs improves fibrosis detection performance and robustness to noise of the MOP-EGM maps, when comparing them to their unaligned versions. The highest ACC was obtained by CV maps computed with the MOP-EGM method in square cliques, including prior alignment of b-EGMs ($CV_{i,j}^{o\text{-}m,a}$), yielding values >90% for all noise levels. On the other hand, CV maps based on the original OP-EGM approach (*CV*^*o*^) proved to be unstable with noise, both in square and triangular clique configurations. As shown in panel **B**, voltage maps performed with the MOP-EGM method, including b-EGMs alignment, and with square cliques, $V_{i,j}^{o\text{-}me,a}$ and $V_{i,j}^{o\text{-}pca\text{-}r,a}$, are the most robust in reproducing the reference unipolar voltage maps in the presence of noise. They achieve Pearson correlation coefficients >0.80 for all noise levels and present smaller interquartile ranges than bipolar maps.

**Figure 10:**
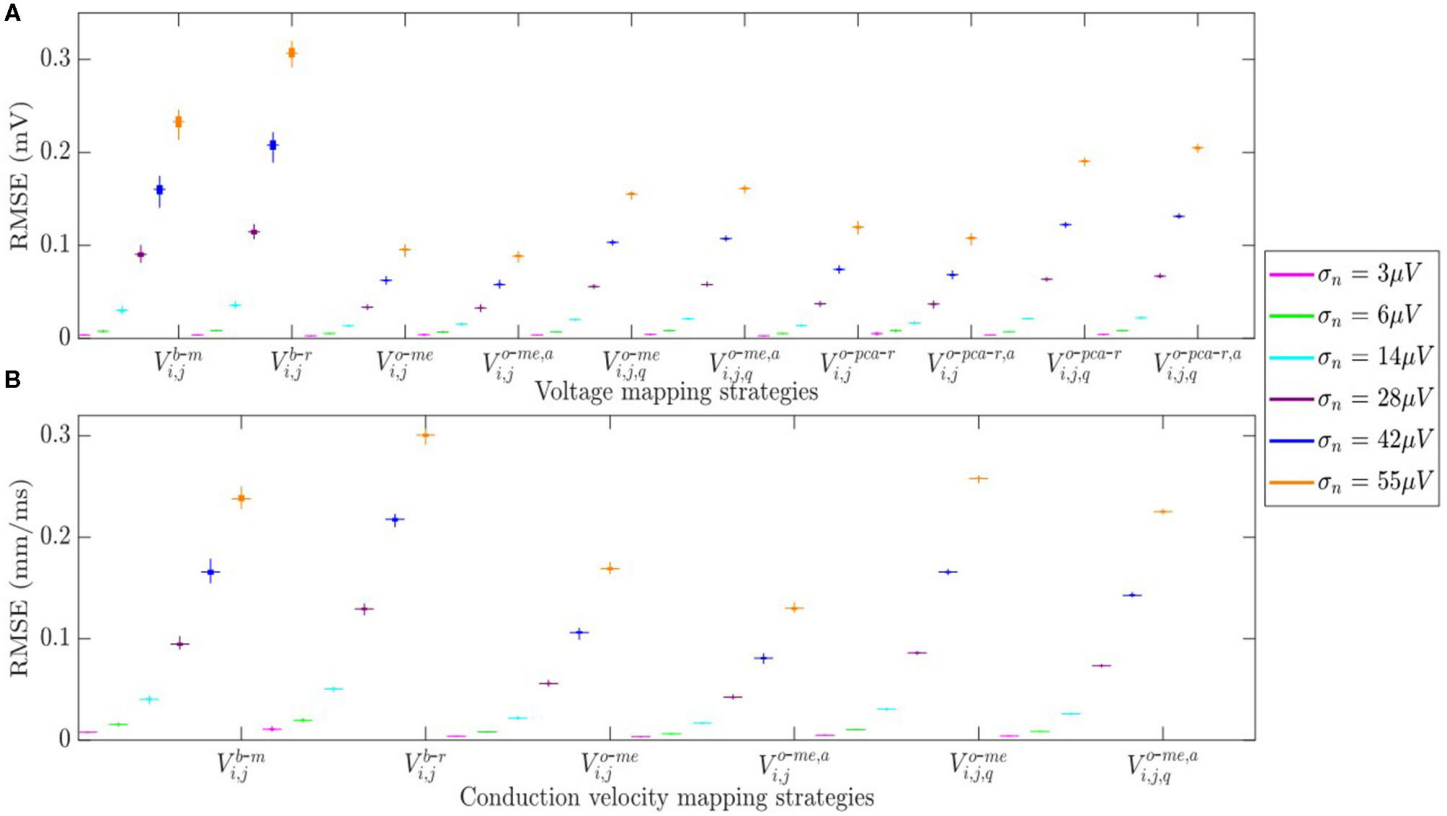
**(A)** RMSE for voltage mapping strategies. **(B)** RMSE for velocity mapping strategies. For each noise level, the central mark and the bottom and top edges of each box indicate the median, the first and the third quartile, respectively.

The RMSE between maps computed from noisy b-EGMS and those computed with noise-free b-EGMs are depicted in Figures 10A,B, respectively, for each noise level. As in previous results, all three MEA orientations are considered jointly. The RMSE values for omnipolar voltage maps are all lower than 0.2 mV, whereas they achieve 0.3 mV in bipolar maps for the highest noise level. As for the CV maps, the MOP-EGM method shows lower RMSE values than the standard OP-EGM approach, in agreement with what was found in Figure 8.

Table 2 reports the mean and standard deviation of the error caused by each noise level in the propagation angles estimated with both omnipolar approaches θ^*o*^ and θ^*o*-*m*^ for square and triangular cliques, considering jointly all three MEA orientations. These results show that propagation direction maps performed with square cliques are less affected by noise than those performed with triangular cliques, regardless of the omnipolar approach. However, for noise levels lower than σ_*n*_ = 28μ*V*, direction maps with the MOP-EGM approach θ^*o*-*m*^ exhibit a smaller mean bias than those using the original method θ^*o*^.

**Table 2:** Expected error ϵ_θ_ (presented as mean ± SD) estimated globally for all propagation direction maps performed with noisy b-EGMs with respect to clean map.

ϵ_**θ**_ **(deg) without || with b-EGMs alignment**				
Map type	Cliques	σ_*n*_ = 3μ*V*	σ_*n*_ = 6μ*V*	σ_*n*_ = 14μ*V*
θ^*o*^	□	0.00 ± 0.18	−0.01 ± 0.32	−0.01 ± 0.72
	△	−0.02 ± 2.86	−0.03 ± 3.74	−0.03 ± 5.10
θ^*o*-*m*^	□	0.00 ± 0.19||−0.01 ± 0.41	0.01 ± 0.34||0 ± 0.64	0.01 ± 0.72||0.03 ± 1.24
	△	0.02 ± 2.76||0 ± 0.52	0.02 ± 3.58||0 ± 0.76	0.08 ± 4.81||0.01 ± 1.65
Map type	Cliques	σ_*n*_ = 28μ*V*	σ_*n*_ = 42μ*V*	σ_*n*_ = 55μ*V*
θ^*o*^	□	0.00 ± 1.38	0.00 ± 2.02	0.00 ± 2.63
	△	−0.05 ± 6.77	0.02 ± 8.14	0.02 ± 9.33
θ^*o*-*m*^	□	0.02 ± 1.35||0.05 ± 2.50	0.01 ± 1.99||0.09 ± 3.86	0.04 ± 2.60||0.22 ± 5.51
	△	0.12 ± 6.31||0.03 ± 3.33	0.17 ± 7.54||0.07 ± 5.24	0.20 ± 8.75||0.17 ± 7.45

**Figure 11:**
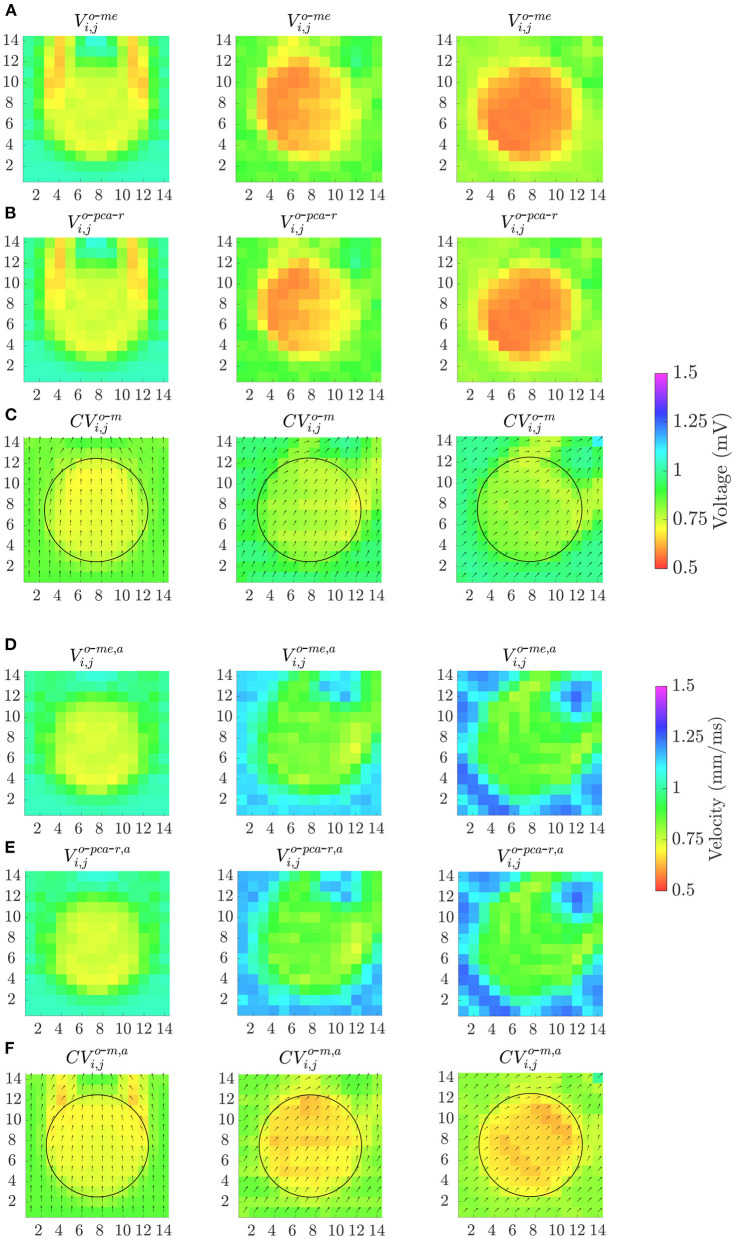
Omnipolar voltage maps **(A,B,D,E)** and velocity maps **(C,F)** performed from clean b-EGMs, without **(A–C)** and with **(D–F)** previous time alignment, by using square cliques, when: Ψ = 0° (left column); Ψ = 30° (middle column) and Ψ = 45° (right column).

Finally, Figure 11 shows the effect of the MEA orientation on omnipolar voltage and velocity maps (*V*^*o*-*me*^, *V*^*o*-*pca*-*r*^, *V*^*o*-*me, a*^, *V*^*o*-*pca*-*r, a*^, *CV*^*o*-*m*^ and *CV*^*o*-*m, a*^) performed with square cliques. The accuracy in fibrotic patch detection reached the values ACC = 91, 99, and 100% using *V*^*o*-*me*^; ACC = 91, 100, and 100% using *V*^*o*-*pca*-*r*^; and ACC = 100, 91, and 91% using *CV*^*o*-*m*^, when Ψ = 0°, Ψ = 30° and Ψ = 45°, respectively. When previous time alignment of b-EGMs is performed, accuracy achieves values ACC = 100, 91, and 92% for both *V*^*o*-*me, a*^ and *V*^*o*-*pca*-*r, a*^, while ACC = 93, 100, and 100% when using *CV*^*o*-*m, a*^, for the same catheter orientations.

## 4. Discussion

In this work, we investigated the performance of different substrate and velocity mapping modalities based on the OP-EGM method, and on the here proposed MOP-EGM approach, in characterizing and detecting fibrotic areas. For that purpose, we used a 2D multi-electrode array over an atrial tissue simulated with the Courtemanche cellular model.

The OP-EGM methodology was presented in Deno et al. (2017) as a novel approach allowing characterization of myocardial substrate and propagation pattern, making possible real-time high-density maps less sensitive to catheter orientation than current bipolar strategies. It showed to solve complex collision and fractionation wavefront patterns in animal subjects, thus providing coherent voltage maps even during atrial fibrillation (Haldar et al., 2017). The OP-EGM approach was proposed to overcome the well-known weaknesses affecting bipolar voltage mapping, which is the cornerstone for the identification of fibrotic atrial substrate, and consequently for low-voltage-guided catheter ablation strategies in AF (Haldar et al., 2017). The efficacy of omnipolar EGMs was also proved in *ex vivo* (Magtibay et al., 2017) and *in vivo* (Porta-Sánchez et al., 2019) ventricular mapping, for delineation of both healthy and infarcted areas. OP-EGM approach-based mapping strategies represent an alternative to the more time-consuming local activation times mapping, allowing beat-to-beat indication of the wavefront propagation direction (Deno et al., 2020). However, we observed in our work that OP-EGM still presents some orientation-dependent behavior affecting fibrosis detection, voltage and CV estimations, and we propose an alternative, referred as MOP-EGM method, partially overcoming these problems.

We started by analyzing maps computed from noise-free b-EGMs. Our results show that the previous alignment of the b-EGMs in each clique (as proposed in the MOP-EGM method) always improves the fibrosis detection ability of the omnipolar voltage and conduction velocity maps. The latter provide a characterization of the atrial substrate comparable to bipolar voltage maps combining b-EGMs along *x* and *y* directions. On the other hand, bipolar maps *V*^*b*-*x*^ and *V*^*b*-*y*^ in each of the MEA main directions revealed ACC values of 69 and 91%, respectively. Moreover, CV maps computed with the MOP-EGM approach present better performance than both omnipolar voltage maps and CV maps computed with the original OP-EGM method. It must also be pointed out that, using the omnipolar approaches, conduction velocities are estimated without the need of detecting local activations. Therefore, the sensitivity to inaccuracies in the determination of local activation times is avoided.

Most studies to detect fibrosis rely on bipolar voltage mapping (Rodríguez-Mañero et al., 2018; Yamaguchi et al., 2019). Other works demonstrate a high correlation with respect to the use of u-EGMs based maps, suggesting no relevant clinical impact of using b-EGMs over u-EMGs (Nairn et al., 2020). Future comparison of unipolar maps with the here proposed MOP-EGM approach will elucidate to what extend that statement remains valid.

The present work also aims at studying the sensitivity to noise of the different mapping approaches. Our observations suggest that the voltage and conduction velocity maps performed from aligned b-EGMs are more robust against noise than both bipolar maps and their unaligned versions, also showing better performance in discriminating fibrosis and in reproducing voltage maps for the same noise level.

In the present study we also addressed the question of the sensitivity of the OP-EGM method to the MEA orientation. Although the fibrotic patch was well discriminated from the healthy tissue by all omnipolar mapping strategies considered in this study, both voltage and velocity OP-EGM based estimates were affected by the relative orientation between MEA and tissue fibers. Regarding voltage maps, when the MEA is not oriented in the same direction as tissue fibers (as when Ψ ≠ 0°), we observe a reduction of the omnipolar voltage values. In addition, in tissue areas where the wavefront propagation is curved, the electric field is overestimated when the local propagation direction becomes parallel to one of the two main directions of the catheter (as when Ψ = 45°). As this happens in an area of healthy tissue, it causes a higher overall accuracy in fibrotic patch detection. The reverse occurs when these curved areas are oblique to the MEA (as in Ψ = 0°), providing underestimated voltages and decreased fibrotic detection accuracy.

As for the orientation dependence of velocity maps, the opposite behavior is observed, with velocity estimation increasing for Ψ ≠ 0°. This is explained by the fact that *v*, in Equation (29), has an inverse relationship with the magnitude of the estimated E-field, while in voltage estimation (equations in subsection 2.9), the dependence is direct. In addition, in those areas where the wave propagation is curved, velocity is overestimated when the MEA is in the same direction as the tissue (Ψ = 0°). Therefore, the fibrotic patch is globally better detected for this configuration. On the other hand, if catheter becomes oblique to the tissue (Ψ ≠ 0°), areas of curved propagation appear underestimated, thus reducing the overall fibrosis detection performance for those configurations. When b-EGMs are time-aligned, both MOP-EGM based voltage and velocity maps continue to be sensitive to the orientation between MEA and tissue, but to a lesser extent if compared to their unaligned versions. As a result, improved fibrosis detection, better voltage estimations and less degradation by noise are observed in average when using the MOP-EGM approach. The remaining dependence with Ψ is not predicted by the mathematical model based on the plane-wave assumption, but the sampling frequency of the b-EGMs (1 kHz) could be behind this observation.

## 5. Limitations

The basic assumption of plane and homogeneous propagation within each clique represents an intrinsic limitation of the omnipolar methodology, requiring the use of high spatial resolution multi-electrode catheters (like the one considered in this study). Moreover, the hypothesis of a plane myocardial surface in the area defined by the clique is questionable in non-compacted myocardial regions or in the atrial appendage. Nevertheless, those areas will be identified with 3D electroanatomic mapping systems (Deno et al., 2017).

In this work, we simulated a very simple propagation pattern in a 2D atrial model, which does not perfectly reproduce the real 3D situation. Therefore, the results here obtained need to be confronted in future studies with those from other simulation configurations (such as non-plane or multiple wavefronts in atrial fibrillation, as well as patchy fibrosis models). Moreover, the proposed MOP-EGM maps need to be evaluated over real data, where the effect of electrode size, inter-electrode distance and variable tissue-electrode contact will be present. The validation of these methods *in-vivo* is, though, challenging, since late gadolinium-enhanced magnetic resonance imaging represents the only non invasive available reference for atrial fibrosis today, and even their utility is under debate (Caixal et al., 2020). *In-vivo*/*Ex-vivo* animal experiments represent an alternative for future works aiming to establish if the simulation-proved superior performance of MOP-EGM translates to clinical counterparts.

## 6. Conclusion

In this work we showed that voltage mapping strategies based on the MOP-EGM method are able to discriminate fibrotic from healthy tissue. For low noise levels, they attain comparable performance to maps obtained by combining the b-EGMs amplitudes along the two directions of the catheter, but clearly outperform bipolar maps when noise is added. On the other hand, omnipolar CV maps reveal higher fibrosis detection accuracy than voltage maps, specially when the modifications proposed in this work were applied. The fibrosis detection performance of voltage and velocity maps benefit from prior intra-clique time alignment of b-EGMs as proposed in the MOP-EGM approach. In addition, the use of square cliques outperforms the triangular ones.

As for the different approaches to estimate the omnipolar voltage from the E-fiel loop, both the one based on maximal excursion (*me*) and the one based on principal component analysis (*pca*) yield similar results, in terms of detection accuracy and correlation with unipolar voltage maps. Similar conclusions are drawn when b-EGMs are affected by noise extracted from real signals.

Both OP-EGM and MOP-EGM strategies considered to estimate the propagation angle revealed smaller sensitivity to noise than methods based on bipolar voltage maps. The use of square cliques results in lower variance than the use of triangular ones when estimating the propagation angles in noisy situations.

## Data Availability Statement

The raw data supporting the conclusions of this article will be made available by the authors, without undue reservation.

## Ethics Statement

The studies involving human participants were reviewed and approved by the Central Lisbon University Hospital Centre (CHULC) Ethics Committee. The patients/participants provided their written informed consent to participate in the study.

## Author Contributions

JR, AA, PL, and JM designed the study and analyzed the results. JR, PL, and JM wrote most of the manuscript. SR, LM-M, and JS performed the electrophysiological simulations. SL provided the clinical data. PL and JM supervised and formalized the project. JR developed the software and performed the required computations. All authors critically revised the manuscript and approved the submitted version.

## Conflict of Interest

The authors declare that the research was conducted in the absence of any commercial or financial relationships that could be construed as a potential conflict of interest.

## Publisher's Note

All claims expressed in this article are solely those of the authors and do not necessarily represent those of their affiliated organizations, or those of the publisher, the editors and the reviewers. Any product that may be evaluated in this article, or claim that may be made by its manufacturer, is not guaranteed or endorsed by the publisher.
