# Supplementary material for: Characterization of Atrial Propagation Patterns and Fibrotic Substrate With a Modified Omnipolar Electrogram Strategy in Multi-Electrode Arrays
Source: Front Physiol. 2021 Sep 3;12:674223. doi: 10.3389/fphys.2021.674223 (PMC8446360; doi:10.3389/fphys.2021.674223)
Supplement: Supplementary file 1 [file Data_Sheet_1.pdf]

Supplementary Material

Fig. S1 shows the action potentials (APs) after 1 minute of stabilization at basic cycle length (BCL) 500 ms. Signals were registered in two different nodes from the mesh: in a myocyte outside the fibrotic patch (in blue and yellow) and in a myocyte inside the fibrotic patch coupled with two fibroblasts (in orange and purple). In order to show the effect of the applied chronic atrial fibrillation (cAF) electrical remodeling, APs were computed in both control and cAF conditions.

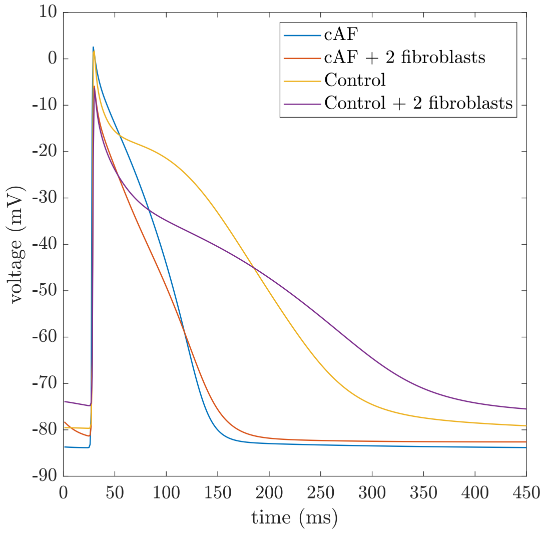

Figure S1. Action potentials produced in control and in chronic atrial fibrillation (cAF) conditions after 1 minute of stabilization at BCL 500 ms.

Table S1 provides variations of the maximum conductances g to reproduce atrial electrical remodeling under cAF conditions.

Table S1. Variation of the maximum conductances g to reproduce atrial electrical remodeling under cAF conditions.

	g_{to}	g_{CaL}	g_{K1}	g_{Kur}	g_{Ks}
**Control**	1.00	1.00	1.00	1.00	1.00
**cAF**	0.25	0.35	2.00	0.55	2.00
**References**	(Caballero et al., 2010)	(Van Wagoner et al., 1999) (Workman et al., 2001)	(Dobrev et al., 2001) (Voigt et al., 2010) (Bosch et al., 1999)	(Caballero et al., 2010)	(Caballero et al., 2010)

Fig. S2 **(A)** shows two of the u-EGMs simulated in this study, $u_{14,2}(t)$ and $u_{14,3}(t)$, computed at electrode sites (14,2) and (14,3) of the MEA, respectively. Resulting b-EGM $b^y_{14,2}(t) = u_{14,3}(t) - u_{14,2}(t)$, derived along y direction of the MEA, is represented in Fig. S2 **(B)**. In Fig. S2 **(C)**, one of the one hundred noisy b-EGMs corresponding to $b^{y,n}_{14,2}(t)$, obtained by randomly adding one of the one hundred different realizations of the recorded noise to $b^y_{14,2}(t)$. is shown for the noise level σ_n = 14 μV. All the signals are plotted only in the activation interval.

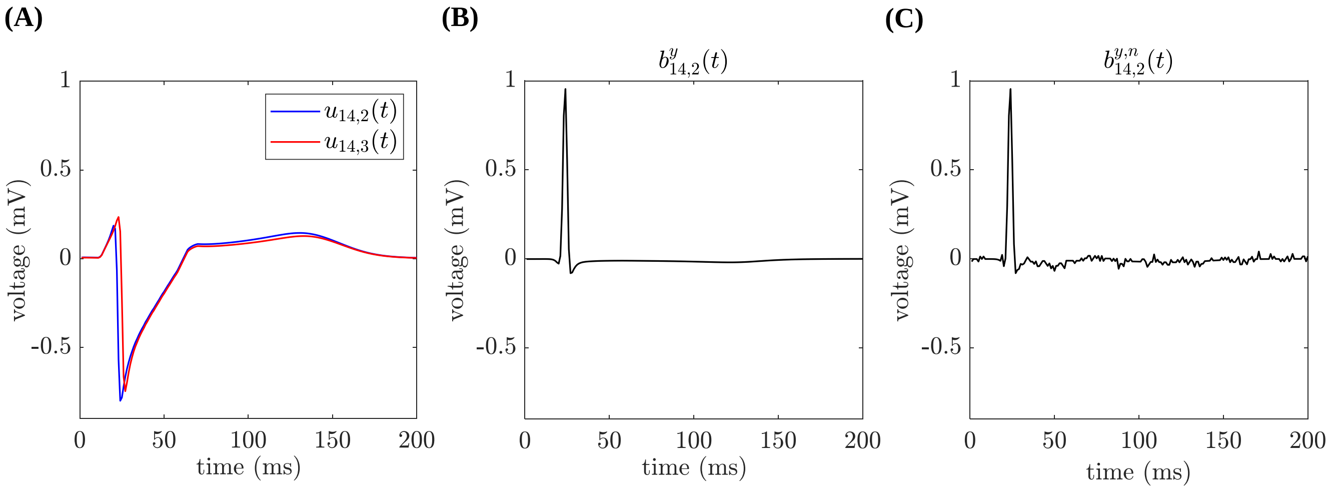

Figure S2. (**A**) Unipolar EGMs $u_{14,2}(t)$ and $u_{14,3}(t)$ and resulting b-EGM $b^y_{14,2}(t) = u_{14,3}(t) - u_{14,2}(t)$, performed without (**B**) and with (**C**) added noise.

REFERENCES

Bosch, R. F., Zeng, X., Grammer, J. B., Popovic, K., Mewis, C., and Kühlkamp, V. (1999). Ionic mechanisms of electrical remodeling in human atrial fibrillation. *Cardiovascular Research* 44, 121–131. doi:10.1016/S0008-6363(99)00178-9

Caballero, R., de la Fuente, M. G., Gómez, R., Barana, A., Amorós, I., Dolz-Gaitón, P., et al. (2010). In humans, chronic atrial fibrillation decreases the transient outward current and ultrarapid component of the delayed rectifier current differentially on each atria and increases the slow component of the delayed rectifier current in both. *J Am Coll Cardiol* . 55, 2346–54. doi:10.1016/j.jacc.2010.02.028

Dobrev, D., Graf, E., Wettwer, E., Himmel, H. M., Hála, O., Doerfel, C., et al. (2001). Molecular basis of downregulation of g-protein-coupled inward rectifying k(+) current (i(k,ach) in chronic human atrial fibrillation: decrease in girk4 mrna correlates with reduced i(k,ach) and muscarinic receptor-mediated shortening of action potentials. *Circulation* 104, 2551–7. doi:10.1161/hc4601.099466

Van Wagoner, D. R., Pond, A., Lamorgese, M., Rossie, S., McCarthy, P., and Nerbonne, J. (1999). Atrial L-type Ca2+ currents and human atrial fibrillation. *Circulation Research* 85, 428–436. doi:10.1161/01.RES.85.5.428

Voigt, N., Trausch, A., Knaut, M., Matschke, K., Varró, A., Wagoner, D. R. V., et al. (2010). Left-to-right atrial inward rectifier potassium current gradients in patients with paroxysmal versus chronic atrial fibrillation. *Circ Arrhythm Electrophysiol* . 3, 472–80. doi:10.1161/CIRCEP.110.954636

Workman, A. J., Kane, K., and Rankin, A. (2001). The contribution of ionic currents to changes in refractoriness of human atrial myocytes associated with chronic atrial fibrillation. *Cardiovascular Research* 52, 226–235. doi:10.1016/S0008-6363(01)00380-7
